# Rapid Evolution of the Embryonically Expressed Homeobox Gene *LEUTX* within Primates

**DOI:** 10.1093/gbe/evad097

**Published:** 2023-05-26

**Authors:** Thomas D Lewin, Josephine R Blagrove, Peter W H Holland

**Affiliations:** Department of Biology, University of Oxford, Oxford, United Kingdom; Department of Biology, University of Oxford, Oxford, United Kingdom; Department of Biology, University of Oxford, Oxford, United Kingdom

**Keywords:** fast-evolving, embryonic genome activation, homeodomain, PRD-class, preimplantation, protein evolution

## Abstract

LEUTX is a homeodomain transcription factor expressed in the very early embryo with a function around embryonic genome activation. The *LEUTX* gene is found only in eutherian mammals including humans but, unlike the majority of homeobox genes, the encoded amino acid sequence is very different between divergent mammalian species. However, whether dynamic evolution has also occurred between closely related mammalian species remains unclear. In this work, we perform a comparative genomics study of *LEUTX* within the primates, revealing dramatic evolutionary sequence change between closely related species. Positive selection has acted on sites in the LEUTX protein, including six sites within the homeodomain; this suggests that selection has driven changes in the set of downstream targets. Transfection into cell culture followed by transcriptomic analysis reveals small functional differences between human and marmoset LEUTX, suggesting rapid sequence evolution has fine-tuned the role of this homeodomain protein within the primates.

**Significance**

Homeobox genes are key regulators of animal development and are therefore often highly conserved between divergent species. However, recent work has uncovered several apparently fast-evolving homeobox genes expressed during mammalian development, one of which is *LEUTX*. Here, we show that the *LEUTX* genes of primates are highly variable, and that these sequence changes have resulted in small modifications to its protein function. This continued rapid divergence of an embryonic gene between closely related species is significant to the areas of evolutionary, developmental, and genome biology.

## Introduction

Homeobox genes are renowned for their conservation across large evolutionary timescales. Many homeodomain (HD) transcription factors (TFs) play essential roles in fundamental animal developmental processes, such as axial patterning, cellular differentiation, and cell proliferation (Duboule 1994; Gehring et al. 1994; Bürglin and Affolter 2016), and have been at the center of the idea of the conserved genetic toolkit due to their striking similarity across widely divergent animal species (Carroll 2000, 2008).

It is therefore intriguing that in recent years, an increasing number of homeobox genes have been found to be lineage specific and rapidly evolving, contrary to the evolutionary conservation typical of this group. This is particularly the case for the PRD-like class of homeobox genes, to which the fast-evolving and mammal-specific Cphx, Dux, Rhox, and Eutherian Totipotent Cell Homeobox (ETCHbox) genes belong. All of these gene groups have been recruited to roles in early mammalian development (Li et al. 2006; Leidenroth and Hewitt 2010; MacLean and Wilkinson 2010; Madissoon et al. 2016; Maeso et al. 2016).

The ETCHbox genes duplicated from the *CRX* homeobox gene in the ancestor of eutherians and the last eutherian common ancestor possessed six group members: *ARGFX*, *DPRX*, *LEUTX*, *PARGFX*, *TPRX1*, and *TPRX2* (Maeso et al. 2016). ETCHbox genes are expressed exclusively during early preimplantation development (Maeso et al. 2016); recent work has shown that they function around or immediately after embryonic genome activation (EGA) in humans and mice, with early transcriptional programs defective when they are knocked down (Töhönen et al. 2015; Jouhilahti et al. 2016; Madissoon et al. 2016; Guo et al. 2022; Zou et al. 2022). Mouse ETCHbox proteins are necessary for proper blastocyst development and hatching (Cui et al. 2016), and we recently showed that bovine ETCHbox proteins have probable roles in blastocyst formation (Lewin et al. 2022). Moreover, the ETCHbox gene *TPRX1* is necessary for transforming pluripotent human embryonic stem cell cultures into totipotent 8-cell-like cells, suggesting a role in totipotency (Mazid et al. 2022).

This body of work implies that ETCHbox proteins are critical regulators of developmental processes in the mammalian preimplantation embryo. The paradox is that, despite these roles, ETCHbox genes seem to be evolving rapidly. ETCHbox repertoires have undergone dramatic evolutionary changes across the eutherians, with high rates of both gene duplications and losses leading to dramatic copy number variation between species (Maeso et al. 2016; Katayama et al. 2018; Lewin et al. 2021). An illuminating comparison is between humans, which have lost just *PARGFX* and have a single copy of the other five genes, and mice, in which *ARGFX*, *DPRX*, *PARGFX*, and *LEUTX* are all lost or pseudogenized, *TPRX1* is present in two copies, and *TPRX2* in 66 copies (Maeso et al. 2016; Royall et al. 2018). Other large tandem arrays of ETCHbox genes have been found in *Oryctolagus cuniculus* (rabbit—27 *LEUTX* copies), *Cavia porcellus* (guinea pig—14 *LEUTX* copies), and *Myotis myotis* (greater mouse-eared bat—six *TPRX2* copies) (Maeso et al. 2016; Lewin et al. 2021). However, previous work employed a broad sampling strategy, leaving open the question of whether closely related species possess different ETCHbox repertoires.

The differences between mammalian taxa are not restricted to gene duplication and loss. ETCHbox genes and their “ancestor” *CRX* exhibit asymmetric sequence evolution: *CRX* has been conserved while ETCHbox sequences have diverged extensively between taxa, and this divergence has been driven at least in part by positive selection (Maeso et al. 2016; Lewin et al. 2021). In previous work, we compared the transcriptional activity of ETCHbox genes between humans, mice, and cattle and found evidence for changes in gene function (Lewin et al. 2022); we define “function” here as the gene sets up- and downregulated by a putative TF.

Overall, previous work has shown that extensive sequence divergence and changes in ETCHbox protein function are seen between deeply diverged evolutionary lineages of eutherian mammals. We asked to what extent there have been changes between more closely related species. This will help answer whether ETCHbox homeobox genes are “fast-evolving” or whether they underwent change during mammalian diversification followed by relative stasis. To address this question, we characterized the ETCHbox gene *LEUTX* across the order primates, with species spanning from a few million years to circa 75 million years of divergence (Wilkinson et al. 2011; Pozzi et al. 2014; Reis et al. 2018). We find that *LEUTX* sequences have continued to diverge at a rapid rate across primates and that positive selection has driven substitutions at key HD residues, suggesting selection for divergence of protein function. Experimental characterization using transfection followed by RNA-sequencing (RNA-seq) suggests small but significant differences exist in the TF function of LEUTX between primate species.

## Results

### Duplication and Divergence of *LEUTX* within Primates

**Fig. 1.**
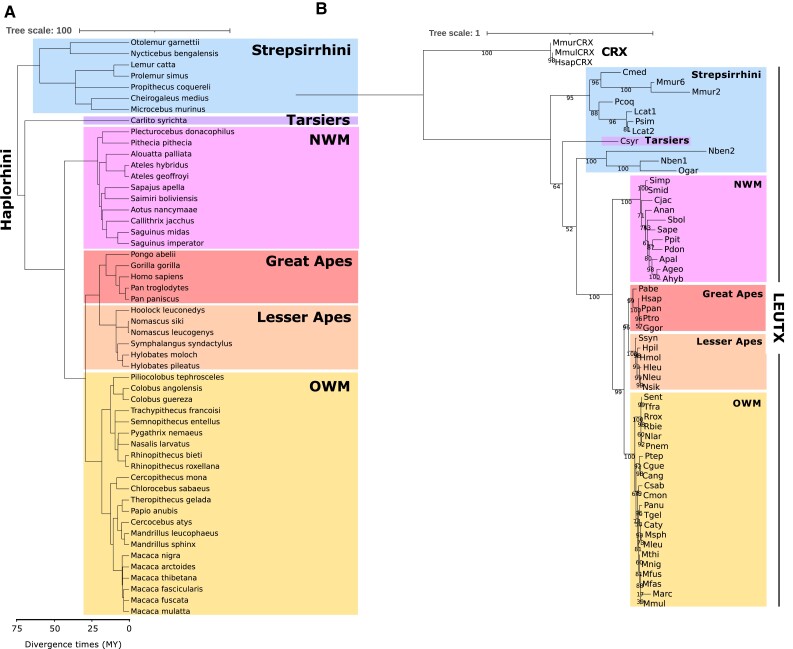
Phylogenetic analysis of primate LEUTX sequences. (*A*) Species tree of the 52 primates used in this analysis. Branch lengths are proportional to estimated divergence times (MY, million years). (*B*) ML tree of primate LEUTX sequences made with full protein-coding sequences (substitution model: JTT + G4). Great ape, lesser ape, Old World monkey, and New World monkey clades are recapitulated with the expected topology. Nben and Ogar are expected to group with the lemurs (Cmed, Mmur, Pcoq, Lcat, and Psim). Ageo, *Ateles geoffroyi*; Ahyb, *Ateles hybridus*; Anan, *Aotus nancymaae*; Apal, *Alouatta palliata*; Cang, *Colobus angolensis*; Caty, *Cercocebus atys*; Cgue, *Colobus guereza*; Cimi, *Cebus imitator*; Cjac, *Callithrix jacchus*; Cmed, *Cheirogaleus medius*; Cmon, *Cercopithecus mona*; Csab, *Chlorocebus sabaeus*; Csyr, *Carlito syrichta*; Ggor, *Gorilla gorilla*, Hleu, *Hoolock leuconedys*; Hmol, *Hylobates moloch*; Hpil, *Hylobates pileatus*; Hsap, *Homo sapiens*; Lcat, *Lemur catta*; Marc, *Macaca arctoides*; Mfasc, *Macaca fascicularis*; Mfus, *Macaca fuscata*; Mleu, *Mandrillus leucophaeus*; Mmul, *Macaca mulatta*; Mmur, *Microcebus murinus*; Mnig, *Macaca nigra*; Msph, *Mandrillus sphinx*; Mthi, *Macaca thibetana*; Nben, *Nycticebus bengalensis*; Nlar, *Nasalis larvatus*; Nleu, *Nomascus leucogenys*; Nsik, *Nomascus siki*; NWM, New World Monkeys; Ogar, *Otolemur garnettii*; OWM, Old World Monkeys; Pabe, *Pongo abelii*; Panu, *Papio anubis*; Pcoq, *Propithecus coquereli*; Pdon, *Plecturocebus donacophilus*; Pnem, *Pygathrix nemaeus*; Ppan, *Pan paniscus*; Ppit, *Pithecia pithecia*; Psin, *Prolemur simus*; Ptep, *Piliocolobus tephrosceles*; Ptro, *Pan troglodytes*; Rbie, *Rhinopithecus bieti*, Rrox = *Rhinopithecus roxellana*; Sape, *Sapajus apella*; Sbol, *Saimiri boliviensis*; Sent, *Semnopithecus entellus*; Simp, *Saguinus imperator*; Smid, *Saguinus midas*; Ssyn, *Symphalangus syndactylus*; Tfra, *Trachypithecus francoisi*; Tgel, *Theropithecus gelada*.

**Fig. 2.**
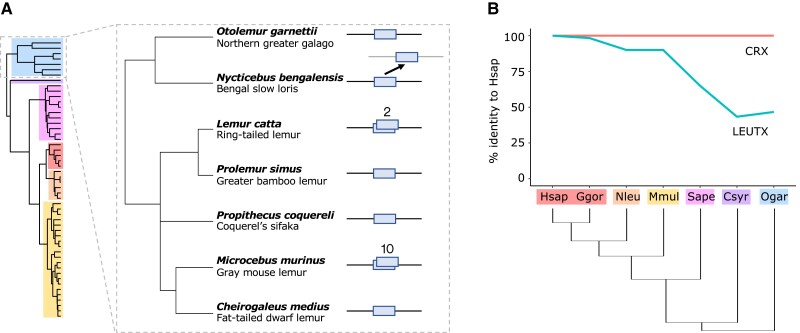
Evolution of LEUTX within primates. (*A*) *LEUTX* copy number within strepsirrhine primates. *N. bengalensis* has a divergent second *LEUTX* copy on a separate scaffold. *L. catta* has two *LEUTX* tandem duplicates, and *Microcebus murinus* has ten *LEUTX* loci. (*B*) Divergence in primate LEUTX HDs. Plot shows percent identity of representative species’ LEUTX HDs to that of human. CRX is shown for reference. Abbreviations as in figure 1.

We identified the *LEUTX* genes in publicly available genome sequences of 52 primate species representing all major evolutionary lineages (fig. 1*A* and *B* and supplementary figs. S1–S3 and table S1, Supplementary Material online). Although *LEUTX* has been lost in several other mammals (Lewin et al. 2021), the gene is present in all of the sampled primate genomes. Of 52 species analyzed, 48 have a single putatively functional *LEUTX* locus in the expected location in the genome. We find four species with duplications: 1) ten *LEUTX* loci in *Microcebus murinus* as reported previously (Lewin et al. 2021); 2) two tandem *LEUTX* loci in *Lemur catta*; 3) a divergent, intron-containing copy on a separate scaffold in *Nycticebus bengalensis*; and 4) a partial gene duplication affecting exons 1 and 3 in *Hylobates pileatus*. The first three examples are all members of the Strepsirrhini, which includes the lemurs and lorises (fig. 2*A*).

We asked whether LEUTX protein sequences are fast-evolving within the primates. Using all versus all pairwise comparisons of sequence identity, we find that primate LEUTX HDs show extensive divergence, increasing gradually with phylogenetic distance (fig. 2*B*). The two most different LEUTX HDs (*Callithrix jacchus* and *M. murinus*) share just 35% sequence identity. Indeed, across the full coding sequence, only 12% (23/198) of amino acid sites are invariable between all sampled primates. Coding sequences are most variable within the Strepsirrhini (figure 1*B* and supplementary table S2, Supplementary Material online). This contrasts markedly with *CRX*, from which *LEUTX* originated by gene duplication, which is highly conserved across species as typical for homeobox genes (fig. 2*B*). Across a sample of 20 species representing all major evolutionary lineages (supplementary fig. S4, Supplementary Material online), 19 of the CRX HDs are identical, while *Pan troglodytes* has one substitution (A18T). Overall, we show that *LEUTX* protein-coding sequences have evolved rapidly within the primate lineage, including within the HD.

The primate *CRX* sequences show no variation in gene structure: the start and stop codons and intron/exon boundaries are conserved, and there are no indels. In contrast, of the 52 *LEUTX* sequences analyzed, there are six different predicted start codon positions and seven different stop codon positions. For example, there are different predicted start codons in Old World monkeys/apes (×2), New World monkeys (×2), tarsiers, and lemurs. This is due to amino acid substitutions and not the shifting of intron/exon boundaries. Additionally, we uncover indels at four separate locations (supplementary fig. S5, Supplementary Material online). Overall, within primates we observe *LEUTX* duplication, rapid sequence evolution, and significant changes to gene structure, but no cases of *LEUTX* gene loss.

Within the genus *Macaca*, we were able to test the extent of variation between very closely related species. Among six species, we find that two have identical deduced LEUTX proteins (*Macaca fascicularis* and *Macaca fuscata*), two differ from this reference by one substitution (*Macaca thibetana* and *Macaca nigra*; both H101R), and one species has a different substitution (*Macaca mulatta*; P92S) (supplementary fig. S6, Supplementary Material online). However, *Macaca arctoides* has 11 amino acid differences: six of these are due to frameshift-causing indel 16 residues from the end of exon 3, changing the frame of the last seven amino acids of the protein and creating a premature stop codon. We find that there is more difference between LEUTX protein sequences within the *Macaca* genus than there is between CRX protein sequences across the entire primate order.

### Evolution of Functional Motifs

**Fig. 3.**
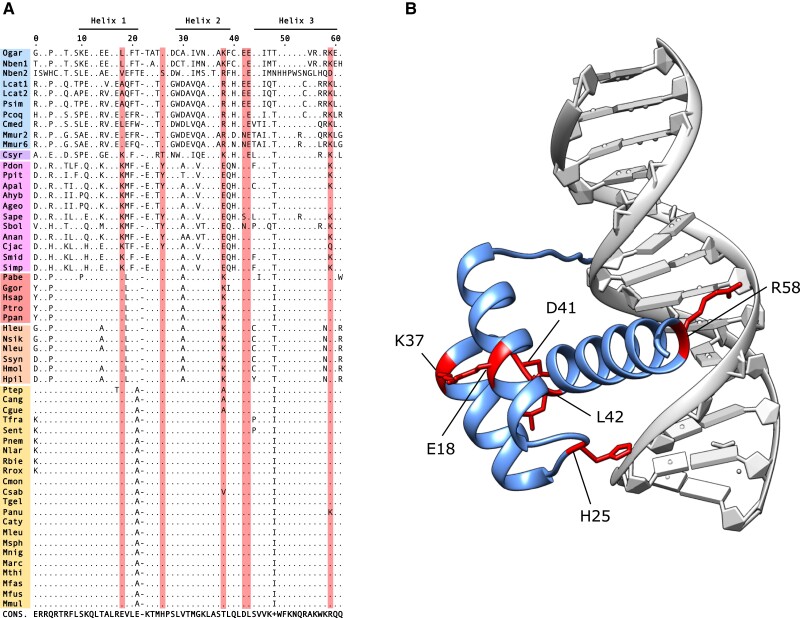
Positive selection in LEUTX HDs. (*A*) Primate LEUTX HDs. Only residues divergent from the consensus are shown; consensus sequence is below alignment. Residues under positive selection are highlighted. Abbreviations as in figure 1. (*B*) Structure of human LEUTX HD (blue) in complex with DNA (gray). Residues at which positive selection was detected in primates are shown in red; side chains shown only for these residues.

We tested whether positive selection has been a driver of LEUTX sequence divergence. We detected evidence for episodic diversifying selection within the primate lineage using the branch–site model of BUSTED (Murrell et al. 2015) (likelihood ratio test [LRT] *P* = 9.322 × 10^−7^). Analysis using MEME (Murrell et al. 2012) indicated that 27 residues within the protein have been under positive selection at some point in the primate phylogeny, six of which lie within the HD (fig. 3*A* and supplementary fig. S5, Supplementary Material online). This suggests that positive selection has played a role in the divergence of LEUTX proteins.

One of the residues inferred to have changed due to positive selection is HD residue 58, found within the critical “recognition helix” (helix 3), which the structural modeling approach of Katayama et al. (2018) identified as a specificity-determining residue in LEUTX. In almost all Old World monkeys and apes (including human), this residue is R58, while the majority of New World monkeys and prosimians (strepsirrhines plus tarsiers) have K58. *C. jacchus* is notable for its unusual Q58 residue. Comparative structural modeling suggests that residue 58 contacts the major groove of the DNA double helix (fig. 3*B*). This suggests that within primates, there has been selection for divergent specificity of LEUTX DNA–binding properties. Modeling also suggests that the side chains of residues under positive selection in HD helices 1 and 2, at positions 18 and 37, respectively, are in close proximity. Their opposite charges and HD position suggest the formation of salt bridges between these residues (Clarke et al. 1994), implying selection for possible changes in the structure or stabilization of the HD. Positively selected residue H25 contacts the DNA sugar–phosphate backbone.

Changes to other specificity-determining residues, as defined by Katayama et al. (2018), have also occurred but are not confirmed as under positive selection with the current data set. First, A54 to V54 in *Otolemur garnettii* and *N. bengalensis* (galago and loris). Second, position 47 has “flipped” between I47 and T47 several times: T47 is seen in prosimians and New World monkeys, changing to I47 in *C. jacchus*; I47 is also seen in Old World monkeys but changed to T47 in the ancestor of apes, again reverting to I47 in *Homo*, *Pan*, and *Gorilla*. This complex evolutionary history suggests lability in this part of the LEUTX protein, consistent with previous work, which found this site has minimal functional influence alone but may undergo compensatory substitutions in response to changes at other positions (Katayama et al. 2018). Other known specificity-determining residues (R2, R3, R5, K50, and N51) are invariant across primates (with the exception of the divergent *N. bengalensis* duplicate), and we identify pervasive purifying selection at R2, R5, and N51, along with 11 other residues within the HD (supplementary fig. S5, Supplementary Material online).

Katayama et al. (2018) annotated a “Leutx domain,” a peptide motif downstream of the HD with conservation across mammals. Within this region, the authors propose two 9-amino acid transactivation domains (9aaTADs) in every mammalian sequence analyzed; 9aaTADs mediate the activation of transcription and are therefore key to TF function (Piskacek et al. 2007). We find both 9aaTADs are highly conserved across the 44 anthropoids analyzed (New World monkeys, Old World monkeys, and apes); we detect evidence for purifying selection at four residues in the first 9aaTAD and two in the second (supplementary fig. S5, Supplementary Material online). As above, increased change is observed within the prosimians.

We also asked whether ubiquitination motifs in LEUTX showed evolutionary conservation across primates. Using an evolutionary screening algorithm (Wang et al. 2017a), we identified three high-likelihood putative ubiquitination motifs in human LEUTX (supplementary fig. S5, Supplementary Material online). Each is conserved across all anthropoids, suggesting evolutionary constraint, but prosimians show notable divergence. For instance, *O. garnettii* is missing the target lysine at two out of three motifs, but putatively compensatory lysine substitutions are present within both of these motifs. Several other species are missing the target lysine in the ubiquitination motifs but evolved new lysine residues elsewhere. The conservation of ubiquitination motifs across anthropoids and the evolution of putative compensatory changes in prosimians points to functional importance, consistent with the genes’ fleeting temporal expression and subsequent requirement for rapid degradation.

### Evolution of LEUTX Expression Profiles

**Fig. 4.**
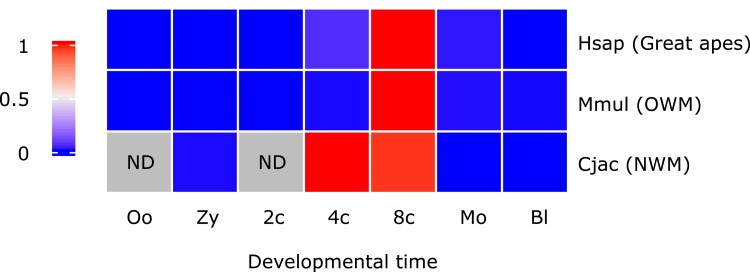
LEUTX expression in primate preimplantation embryos. Heatmap of scaled *LEUTX* expression in human (Hsap), rhesus macaque (Mmul), and common marmoset (Cjac) preimplantation embryos. Gray squares indicate no data available. 2*c*, two-cell; 4*c*, four-cell; 8*c*, 8-cell; Bl, blastocyst; Cjac, *Callithrix jacchus*; Hsap, *Homo sapiens*; Mmul, *Macaca mulatta*; Mo, morula; ND, no data; NWM, New World monkeys; Oo, oocyte; OWM, Old World monkeys; Zy, zygote.

We asked whether the expression profiles of *LEUTX* in the preimplantation embryo are conserved across primates. Human *LEUTX* is expressed in a distinct temporal pattern, with expression peaking sharply at the 8-cell stage (Maeso et al. 2016). We quantified *LEUTX* expression across preimplantation development in publicly available human, *M. mulatta* (Old World monkey) and *C. jacchus* (New World monkey) RNA-seq data sets and found strong conservation of 8-cell stage-specific expression between human and *M. mulatta* (fig. 4 and supplementary table S3, Supplementary Material online). In *C. jacchus*, *LEUTX* is expressed in a more protracted pulse comprising both the 4-cell and 8-cell stages, which may reflect differences in the timing of EGA. Overall, *LEUTX* expression profiles can vary but remain constrained within the limits of the cleavage stages of preimplantation development.

### Evolutionary Divergence of LEUTX Downstream Targets

**Fig. 5.**
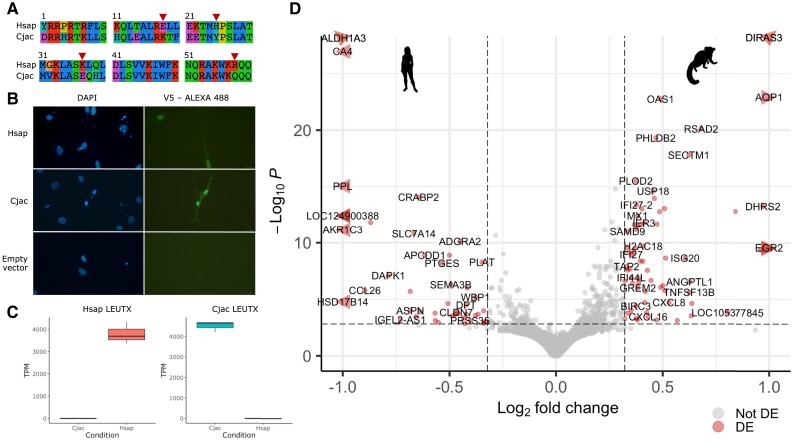
Ectopic expression of human and marmoset *LEUTX* genes. (*A*) Alignment of LEUTX HD sequences of the species used in ectopic expression experiments. Arrowheads mark residues under positive selection with a substitution between human and marmoset. (*B*) Immunocytochemistry of fibroblasts transfected with human and marmoset *LEUTX* genes. DAPI stains DNA blue in cell nuclei. Expression of V5-tagged LEUTX proteins is detected with anti-V5 primary antibodies and Alexa Fluor 488 (green fluorescence)-labeled secondary antibodies. Empty vector transfections showed no green fluorescence. (*C*) Expression of transfected *LEUTX* genes in cell culture samples. (*D*) Transcriptional response to expression of human and marmoset *LEUTX* genes. Points to the left of the center are genes more highly expressed in response to human *LEUTX* expression than marmoset; points to the right are genes more highly expressed in response to marmoset *LEUTX* expression. DE genes (adjusted *P* < 0.05 and fold change > 1.25) are labeled with gene IDs and shown in red. Cjac, *Callithrix jacchus*; DE, differentially expressed; Hsap, *Homo sapiens*; TPM, transcripts per million.

We hypothesized that the selection-driven sequence divergence observed between primates has caused divergence of LEUTX protein functions. We used transcriptome analysis after transfection into cultured cells to test this, targeting *Homo sapiens* (representing great apes) and the common marmoset *C. jacchus* (New World monkeys) for experimental comparison. The *C. jacchus* LEUTX HD has 73% sequence identity to human, including substitutions at four sites within the HD at which we identified positive selection, one of which is the specificity-determining residue 58 (fig. 5*A*). *LEUTX* gene sequences of *H. sapiens* and *C. jacchus*, each with a C-terminal V5 tag, were cloned into a constitutive mammalian expression vector and transfected into human dermal fibroblasts (HDFs). Previous work has shown that expression of ETCHbox genes in a cell culture setting, including in fibroblasts, elicits changes to the expression of embryonic genes (Jouhilahti et al. 2016; Madissoon et al. 2016; Maeso et al. 2016; Royall et al. 2018; Lewin et al. 2021). Immunocytochemistry confirmed protein expression and nuclear localization of the HD TF in both human and marmoset-transfected samples (fig. 5*B*).

RNA-seq was performed on three biological replicates for human and marmoset *LEUTX*, and gene expression was then quantified with Kallisto (Bray et al. 2016) (supplementary table S4, Supplementary Material online). Human (mean transcripts per million [TPM] = 3797) and marmoset (mean TPM = 4514) *LEUTX* genes were successfully expressed in the expected samples (fig. 5*C*). Differential expression analysis was performed to identify differences in the downstream genes responding to human versus marmoset *LEUTX*. We found that expression of human and marmoset *LEUTX* elicited small but notable differences in the transcriptomic response within the transfected cells: 68 genes were more highly expressed in the marmoset-transfected samples, and 44 more highly expressed in the human-transfected samples (fig. 5*D* and supplementary tables S5 and S6, Supplementary Material online). Previous work found expression of human LEUTX to downregulate 754 and upregulate 481 genes (Maeso et al. 2016); this suggests that approximately 9% of the transcriptomic response to human LEUTX is different when marmoset LEUTX is expressed.

**Fig. 6.**
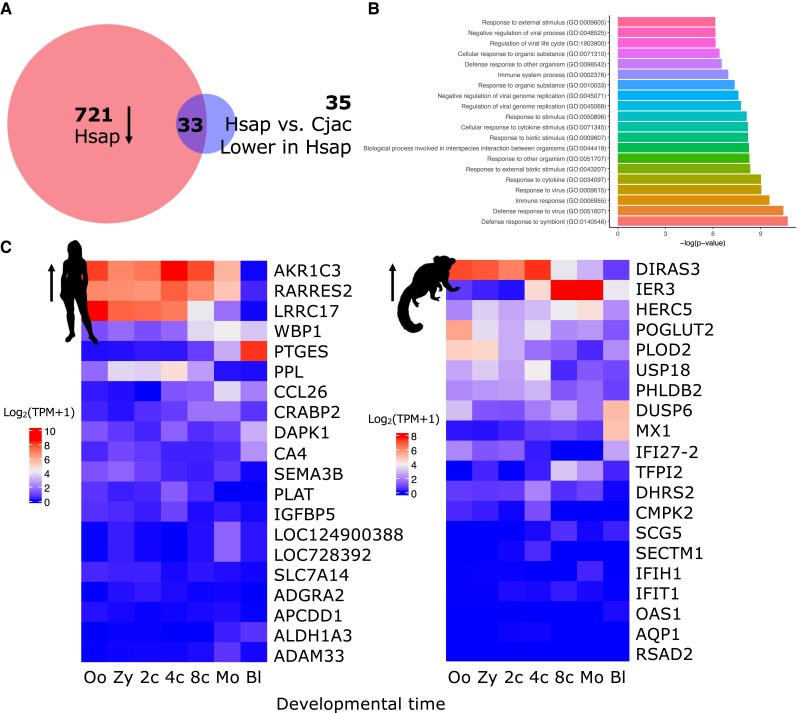
Differing transcriptional responses to human and marmoset LEUTX expression. (*A*) Of the 68 genes more highly expressed in the marmoset LEUTX treatment compared to human LEUTX, 33 are known to be downregulated by human LEUTX. This suggests that they are not (or significantly less) downregulated by marmoset LEUTX. (*B*) Top 20 GO terms enriched in the gene set upregulated in response to marmoset LEUTX compared to human LEUTX. (*C*) Expression of DE genes in the human preimplantation embryo. Left heatmap shows top 20 genes upregulated in response to human LEUTX compared to marmoset, and right heatmap shows top 20 genes upregulated in response to marmoset LEUTX compared to human. 2*c*, two-cell; 4*c*, four-cell; 8*c*, 8-cell; Bl, blastocyst; Cjac, *Callithrix jacchus*; Hsap, *Homo sapiens*; Mo, morula; Oo, oocyte; TPM, transcripts per million; Zy, zygote.

We sought to understand the significance of these transcriptional differences. We find that of the 68 genes more highly expressed in the marmoset treatment compared to human treatment, 33 were previously shown to be downregulated by human LEUTX (Maeso et al. 2016) (fig. 6*A*). This suggests that some genes downregulated by human LEUTX are not downregulated (or significantly less so) by marmoset LEUTX, revealing a change in TF function. We performed biological process Gene Ontology (GO) analysis on these 68 DE genes: all of the top 20 GO terms without exception relate to the response to external biotic stimuli (fig. 6*B* and supplementary table S7, Supplementary Material online). These terms do not appear in the gene set more highly expressed in response to human *LEUTX* than marmoset (supplementary table S8, Supplementary Material online).

We also explored the genes with the strongest difference in response to ectopic expression of human and marmoset *LEUTX*. This allowed us to test whether the genes identified by the ectopic expression approach are realistic embryonic targets of *LEUTX*. We profiled the temporal expression of the top 20 DE genes using published transcriptomic data (Yan et al. 2013) spanning human preimplantation development (fig. 6*C* and supplementary table S9, Supplementary Material online). Ten of the top 20 genes upregulated in the human treatment compared to marmoset are expressed (TPM > 2 in at least one embryonic stage) during preimplantation development. Similarly, 12 of the 20 genes most differentially upregulated in response to marmoset LEUTX are embryonic genes. In addition, several of the most highly expressed DE genes (*Aldo-keto reductase family 1 member C3* [*AKR1C3*], *Retinoic acid receptor responder 2* [*RARRES2*], *Leucine rich repeat containing 17* [*LRRC17*], and *DIRAS family GTPase 3* [*DIRAS3*]) are strongly downregulated around the 8-cell to early morula stage, consistent with the timing of *LEUTX* expression. This suggests that the downstream targets differentially affected by marmoset and human LEUTX are realistic in vivo targets of this HD protein.

Overall, there are clear, significant but relatively minor differences in the downstream targets of human and marmoset LEUTX, suggesting that the evolution of *LEUTX* sequences within the primate lineage has served to subtly modify the proteins’ TF function rather than elicit dramatic shifts in target gene sets.

## Discussion

Fast-evolving homeobox genes may have received less attention than their highly conserved counterparts, but it is becoming increasingly clear that they play important roles in early embryonic development in mammals (MacLean and Wilkinson 2010; Niu et al. 2011; Madissoon et al. 2016; Maeso et al. 2016; Holland et al. 2017). One group with known roles in preimplantation development is ETCHbox, a set of genes in which the copy number, protein-coding sequence, and protein functions have been shown to vary greatly between mammalian taxa (Maeso et al. 2016; Royall et al. 2018; Lewin et al. 2021). In this work, we characterized one of the ETCHbox genes within one taxonomic order, the primates, examining the copy number, amino acid sequence evolution, and divergence of protein function.

Comparative genomic analysis revealed that rapid evolution of the LEUTX protein-coding sequence has occurred to a remarkable extent within the primate lineage. While the CRX HD has remained almost completely unchanged, LEUTX has undergone divergence between primate clades, resulting in an amino acid sequence identity of only 35% between the two most divergent LEUTX HDs in our study, and an average of 70% across all sampled HDs. Positive selection acting on *LEUTX* sequences played an important role in this divergence, acting on key residues across the protein, including six within the HD. The most notable target of positive selection is residue 58, known to be a factor in determining the specificity of LEUTX proteins (Katayama et al. 2018), suggesting there has been selection for changes in protein targets.

Structural modelling revealed that targets of positive selection at HD positions 18 and 37 are positioned on the exterior of their respective helices. A network of salt bridges is known to form between the surfaces of helices 1 and 2 to stabilize the HD structure (Clarke et al. 1994). Salt bridges are bonds between oppositely charged glutamic acid or aspartic acid (negatively charged) and arginine or lysine (positively charged) residues which contribute to protein structure, stability, and specificity (Bosshard et al. 2004; Donald et al. 2011). In the human LEUTX protein, the residues at positions 18 and 37 are glutamic acid and lysine, respectively; this suggests that selection for modifications to salt bridge formation has occurred within primates. Previous work sampling mammals more widely also found positive selection at these residues (Lewin et al. 2021), suggesting that they have been consistent targets for selection across the Eutheria. In addition to selection, it is possible that an elevated mutation rate has contributed to the rapid evolution of *LEUTX*. One potential factor is that genes are more vulnerable to DNA damage at times when they are highly transcribed (Marnef et al. 2017), and DNA damage repair mechanisms are impaired in the cleavage stage embryo when *LEUTX* transcription peaks (Wyatt et al. 2023). However, this is unlikely to be the primary explanation since not all genes expressed in this time period show high rates of sequence change.

*LEUTX* is not lost or pseudogenized in any of the sampled primate species, implying selection for its retention. Although a small number of duplications are observed, these are almost entirely limited to the Strepsirrhini. The relative stability of the *LEUTX* copy number within primates is a notable contrast to the situation across mammals more widely, in which this gene has been lost on at least four independent occasions and has duplicated in multiple species (Lewin et al. 2021). It is enlightening to compare the scenario of *LEUTX* with that of the Reproductive homeobox (Rhox) and Double homeobox (Dux) gene families. Both Rhox and Dux families are PRD-like genes which are mammal-specific, expressed during early development and have rapidly evolving sequences (MacLean et al. 2005; Leidenroth and Hewitt 2010; MacLean and Wilkinson 2010; Eidahl et al. 2016). Like *LEUTX*, the *RHOXF2* protein-coding sequence has diverged rapidly between primates, and copy number variation facilitated by nearby endogenous retroviral sequences also exists between closely related species, such as the presence of two copies in humans and six in chimpanzees (Niu et al. 2011). From Dux genes, we learn that the presence of rapid evolutionary change does not indicate a lack of functional importance, as mouse Dux and its human orthologue DUX4 are both central to EGA despite minimal sequence conservation (Peaston et al. 2004; Macfarlan et al. 2012; Eidahl et al. 2016; De Iaco et al. 2017; Hendrickson et al. 2017; Vuoristo et al. 2022; Yoshihara et al. 2022). The parallels between these three fast-evolving gene families support the idea that selection pressures are acting to drive the evolutionary divergence of groups of homeobox genes with key roles in preimplantation development.

Bioinformatic analyses can reveal evolutionary constraint and the action of positive selection but do not alone reveal the functional consequences of these changes. Using ectopic expression in primary cells, we compared the downstream actions of human LEUTX to the orthologous gene in the common marmoset *C. jacchus*. Differential expression analysis revealed that expression of human and marmoset LEUTX proteins elicits small but notable differences in transcriptomic response. While this stands in stark contrast to the striking differences observed in the function of ARGFX when it was compared across a larger phylogenetic distance between human and cattle (Lewin et al. 2022), it suggests that positive selection has driven minor but detectable changes in LEUTX target specificity between primate species.

What explains this divergence of protein function? LEUTX is a TF activated at EGA with expression at a critical point of mammalian embryonic development (Jouhilahti et al. 2016). At a molecular level, the gene regulatory networks (GRNs) underlying early preimplantation development at the time of, and immediately following, *LEUTX* expression are largely similar across primates but do exhibit small differences (Nakamura et al. 2016; Wang et al. 2017b; Hu et al. 2021). For instance, expression of factors forming the core pluripotency network of the epiblast (NANOG, POU5F1, and SOX2) is conserved between human and marmoset, but further epiblast-specific factors such as CREB3L1, HEY2, INSR, and VENTX are species specific (Boroviak et al. 2018). Overall, the relatively minor differences in LEUTX function between human and marmoset are consistent with the small-scale divergence of the GRNs coordinating preimplantation development; this suggests that positive selection on LEUTX proteins is fine-tuning their roles, changing targets at the periphery of largely conserved GRNs rather than initiating whole-scale changes to the core factors. The observed small differences in timing of LEUTX expression, which is highly specific to the 8-cell stage in humans but expressed in both 4-cell and 8-cell blastomeres in the marmoset, also support the conclusion that rapid sequence evolution has driven small functional adjustments within the primate order. However, such adjustments should not be disregarded as superficial; early development in human and marmoset does indeed entail notable differences, including the duration of preimplantation development and the manner of implantation (Carter and Enders 2004; Boroviak et al. 2018; Siriwardena and Boroviak 2022).

### Conclusions

*LEUTX* is a fast-evolving homeobox gene recruited to a role in EGA in the early mammalian embryo. We characterized the *LEUTX* loci of all available chromosome-level primate genome assemblies, revealing dramatic divergence of protein-coding sequences but limited copy number variation. This divergence has been driven at least in part by positive selection, and six residues in the LEUTX HD were identified as targets of selection within the primate lineage. Ectopic expression experiments suggest that evolutionary sequence change has led to a small divergence in LEUTX function between primate species.

## Materials and Methods

### Comparative Genomics

All reference assemblies of primates with a scaffold N50 of at least 1 Mb were downloaded from NCBI Genome (www.ncbi.nlm.nih.gov/genome/), with selected other species added to improve taxon representation (supplementary table S1, Supplementary Material online). *LEUTX* genes were identified using blastn and tblastn searches and synteny; gene trees and reciprocal blast searches were used to confirm gene identities. The full human *LEUTX* sequence determined from transcriptome data (Maeso et al. 2016) was used as the basis for inferring gene structures. Genes with a complete HD are considered putatively functional. Intronless genes (putative retrocopies) are likely to be nonfunctional due to the absence of regulatory elements (Hurles 2004) and are therefore excluded. In three species, *O. garnettii*, *N. bengalensis*, and *Carlito syrichta*, we were unable to identify the first exon of *LEUTX*. Full LEUTX sequences are available as supplementary figure S7, Supplementary Material online.

For phylogenetics, the maximum likelihood (ML) algorithm of IQ-TREE (Nguyen et al. 2015) was run with 1000 bootstraps made using UFBoot2 (Hoang et al. 2018) and automated model selection by ModelFinder (Kalyaanamoorthy et al. 2017). Sequence alignments were made using Clustal Omega (Sievers et al. 2011) implemented in Seaview version 4.7 (Gouy et al. 2010). A species tree was made using TimeTree 5, which uses a global time-calibrated tree of life synthesized from 4,075 studies (Kumar et al. 2017, 2022). HD sequences of PRD-class proteins were obtained from HomeoDB (Zhong et al. 2008; Zhong and Holland 2011).

Branch–site unrestricted statistical test for episodic diversification (BUSTED) (Murrell et al. 2015) was used to test whether positive selection has acted on *LEUTX* within the primates. The mixed effects branch–site model of MEME (Murrell et al. 2012) was then used to infer sites at which positive selection has acted, and the fixed effects likelihood (FEL) model used to identify pervasive purifying selection (residues where purifying selection is detectable across the whole tree) (Kosakovsky Pond and Frost 2005). Tests for selection were run with default parameters using Datamonkey (Weaver et al. 2018). Where species have a *LEUTX* duplication, only one gene was used in the tests. *Cercopithecus mona* and *Chlorocebus sabaeus* sequences were included up to the ancestral start codon even though this has been lost; their complete HD suggests them to be functional.

The protein structure of the LEUTX HD was modeled by comparative structural modeling using UCSF Chimera 1.16 (Pettersen et al. 2004) to implement Modeller (Šali and Blundell 1993). The *Drosophila melanogaster* Aristaless (Al) HD (PRD-class) in complex with DNA (RCSB Protein Data Bank entry 3LNQ) (Berman et al. 2000; Miyazono et al. 2010) was taken as a reference. Putative ubiquitination sites were detected with ESA-UbiSite (Wang et al. 2017a). HD residues were excluded as potential sites of ubiquitination.

For expression analysis, raw RNA-seq reads from human (*H. sapiens*; PRJNA153427) (Yan et al. 2013), rhesus macaque (*M. mulatta*; PRJNA401876) (Chitwood et al. 2017), and common marmoset (*C. jacchus*; PRJEB29285) preimplantation embryos were obtained from NCBI BioProject (www.ncbi.nlm.nih.gov/bioproject/). *LEUTX* expression was quantified at each developmental stage using Kallisto version 0.48.0 (Bray et al. 2016). Heatmaps were made using ComplexHeatmap version 2.8.0 (Gu et al. 2016) in R version 4.1.0 (R Core Team 2021).

### Ectopic Expression

Primary HDFs (Stemnovate #SV-HF21-17-500) were maintained in HDF medium at 37 °C with 5% CO_2_ and passaged at approximately 70% confluency every 3–4 days. HDF medium consists of Dulbecco's modified Eagle medium (Gibco #41965039) with 10% heat-inactivated fetal bovine serum (Gibco #10500064) and 1% penicillin–streptomycin (Gibco #15140122). Testing for mycoplasma (Sigma Aldrich #MP0035) revealed no contamination.

Codon-optimized sequences of *H. sapiens* and *C. jacchus LEUTX* with a GGGGSGGGGS linker and C-terminal V5 tag (supplementary fig. S8, Supplementary Material online) were synthesized by ThermoFisher GeneArt and cloned into a pcDNA3.1 mammalian expression vector. For transfection, 65,000 cells per well were seeded into 6-well plates. After 16 hours (h), medium was replaced with 2 ml antibiotic-free HDF medium. For each biological replicate, 108 *μ*l Opti-MEM (Gibco #31985-062) was combined with 9.6 *μ*l FUGENE 6 (Promega #E2691) and incubated for 5 min; then, 2.4 *μ*l of 1 *μ*g/*μ*l appropriate expression construct was added before another 15-min incubation. To each well, 120 *μ*l of this mixture was added and cells were kept at 37 °C with 5% CO_2_. After 24 h, transfection medium was removed and replaced with 2 ml HDF medium with 800 *μ*g/ml G418 selective antibiotic (Gibco #10131-035). At 48 h post-transfection, RNA was extracted using an RNeasy Plus Micro kit (Qiagen #74034), and integrity was tested using an Agilent 2100 Bioanalyzer.

To confirm expression of full-length proteins, the immunocytochemistry protocol of Maeso et al. (2016) was used with minor modifications: primary antibody (V5 tag monoclonal antibody; Invitrogen #37-7500) 1:500, 4 h incubation; secondary antibody (goat anti-mouse IgG H + L superclonal recombinant secondary antibody with Alexa Fluor 488; and Invitrogen #A28175) 1:1000, 1 h incubation. Cells were incubated with DAPI (Invitrogen #S36938) to label nuclei. Results were visualized with an Olympus CKX53 inverted fluorescence microscope.

### Analysis of RNA-seq Data

Three replicates for each treatment were sequenced on the Illumina NovaSeq 6000 platform (Novogene). FastQC version 0.11.8 (Andrews 2010) and MultiQC version 1.8 (Ewels et al. 2016) were used for quality control, and reads (150 bp paired-end) were subjected to filtering to remove adapter-containing reads, low-quality reads (*Q* score < 5), and reads with >10% Ns (undetermined bases), resulting in an average of 45.8 million reads per sample. Pseudoalignment to the human transcriptome from genome build GRCh38.p14 (RefSeq annotation) was performed with Kallisto version 0.48.0 (Bray et al. 2016); pseudoalignments were found for an average of 93.8% of reads. Gene-level transcript abundance estimates were created using tximport version 1.20.0 (Soneson et al. 2016) and then differential expression analysis was completed in DESeq2 version 1.32.0 (Love et al. 2014) using apeglm (Zhu et al. 2019) for log fold change (LFC) shrinkage. EnhancedVolcano (Blighe et al. 2022) version 1.16.0 was used to create volcano plots. Genes with an adjusted *P* < 0.05, fold change > 1.25, and mean TPM > 2 were considered differentially expressed. To check whether differentially expressed genes represented realistic embryonic targets, raw reads from human preimplantation development (PRJNA153427) (Yan et al. 2013) were quantified with Kallisto as above (Bray et al. 2016). GO analysis was performed using PANTHER version 17.0 (Thomas et al. 2022) with Fisher's exact test and a false discovery rate (FDR) correction of 0.05.

## Supplementary Material

evad097_Supplementary_Data

## Data Availability

Raw and processed sequencing data sets are available from the NCBI Gene Expression Omnibus (www.ncbi.nlm.nih.gov/geo) under accession GSE224384. Other data underlying the results published in this article are available within its electronic supplementary material.
