# Supplementary material for: Rapid Evolution of the Embryonically Expressed Homeobox Gene LEUTX within Primates
Source: Genome Biol Evol. 2023 May 26;15(6):evad097. doi: 10.1093/gbe/evad097 (PMC10246821; doi:10.1093/gbe/evad097)
Supplement: evad097_Supplementary_Data [file evad097_supplementary_data.zip › Lewin et al. Supplementary Figures S1 - S8.pdf]

Rapid evolution of the embryonically-expressed homeobox gene *LEUTX* within primates

Thomas D. Lewin, Josephine R. Blagrove and Peter W. H. Holland

Electronic Supplementary Material: Supplementary Figures S1 – S8.

Figure S1

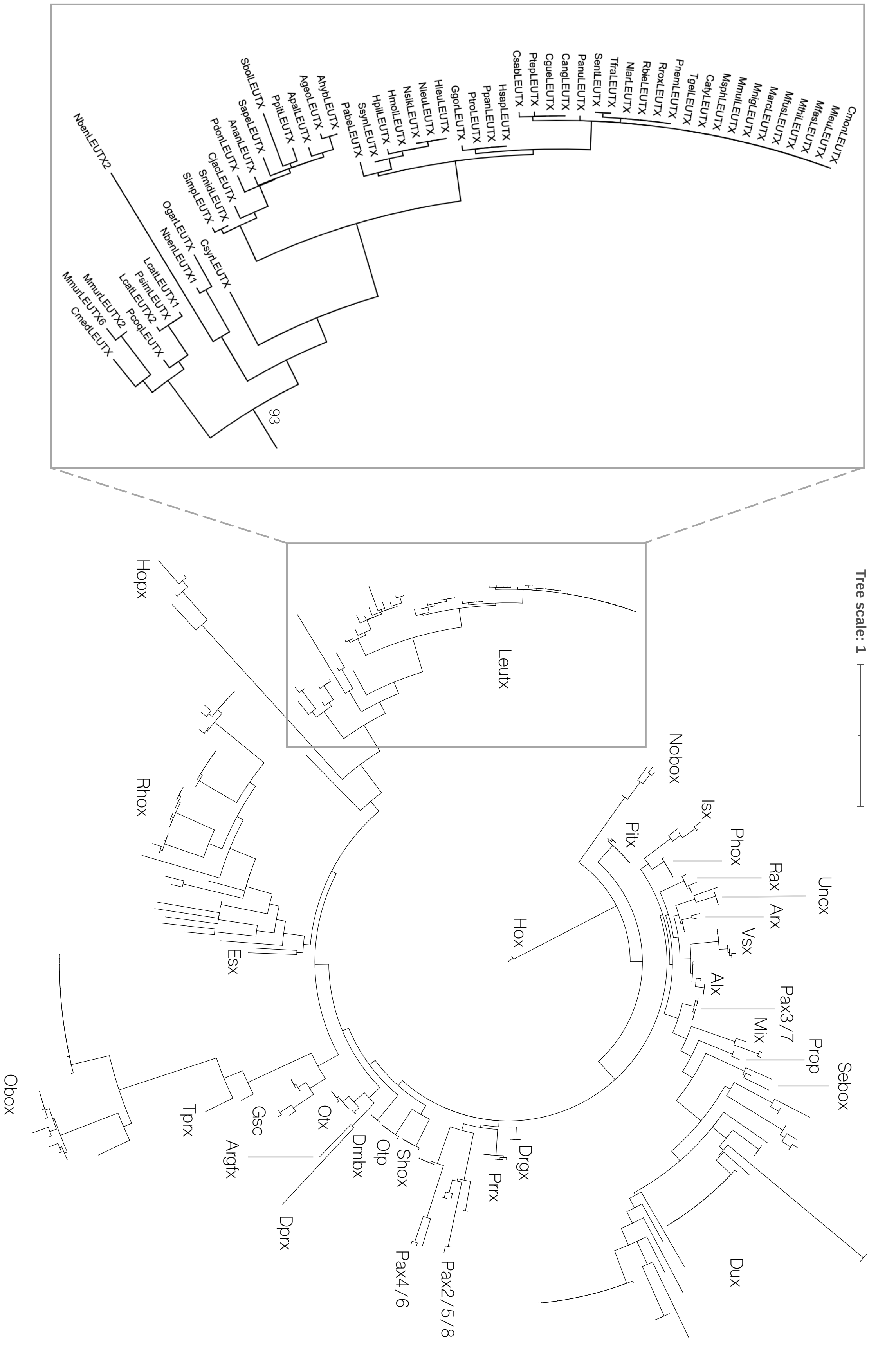
CmonLEUTX
MleuLEUTX
MfasLEUTX
MthiLEUTX
MfusLEUTX
MarcLEUTX
MnigLEUTX
MmulLEUTX
MsphLEUTX
CatyLEUTX
TgelLEUTX
PnemLEUTX
RroxLEUTX
RbieLEUTX
NlarLEUTX
TfraLEUTX
SentLEUTX
PanuLEUTX
CangLEUTX
CgueLEUTX
PtepLEUTX
CsabLEUTX
HsapLEUTX
PpanLEUTX
PtroLEUTX
GgorLEUTX
HleuLEUTX
NsikLEUTX
NleuLEUTX
HmolLEUTX
HpilLEUTX
SsynLEUTX
PabeLEUTX
AhybLEUTX
AgeoLEUTX
ApalLEUTX
PpilLEUTX
SbolLEUTX
SapeLEUTX
AnanLEUTX
PdonLEUTX
CjacLEUTX
SmidLEUTX
SimpLEUTX
NbenLEUTX2
OgarLEUTX
CsyrLEUTX
NbenLEUTX1
LcatLEUTX1
PsimLEUTX
LcatLEUTX2
PcoqLEUTX
MmurLEUTX2
MmurLEUTX6
CmedLEUTX
93
Tree scale: 1
Hopx
Leutx
Rhox
Esx
Obox
Tprx
Gsc
Otx
Argfx
Dmbx
Otp
Shox
Dprx
Pax4/6
Pax2/5/8
Prrx
Drgx
Hox
Nobox
Pitx
Isx
Phox
Rax
Uncx
Arx
Vsx
Alx
Pax3/7
Mix
Prop
Sebox
Dux

Figure S1 Position of LEUTX sequences within a tree of all PRD-class homeodomains. Maximum likelihood (ML) tree built with IQ-Tree (substitution model: JTT+G4). Species name abbreviations as in Figure 1 main text. The alignment used to create this tree is available as Figure S2.

Figure S2

```
                               1
Human|HOXA1|Hox1|ANTP          PNAVRTNFTT KQLTELEK-E FH-FNKYLTR ARRVEIAASL QLNETQVKIW FQNRRMKQKK RE
Mouse|Hoxa1|Hox1|ANTP          PNAVRTNFNT KQLTELEK-E FH-FNKYLTR ARRVEIAASL QLNETQVKIW FQNRRMKQKK RE
Chicken|HOXA1|Hox1|ANTP        ---VRTNFTT KQLTELEK-E FH-FNKYLTR ARRVEIAASL QLNETQVKIW FQNRRMKQKK RE
Frog|hoxa1|Hox1|ANTP           PNTARTNFTT KQLTELEK-E FH-FNKYLTR ARRVEIAAAL QLNETQVKIW FQNRRMKQKK RE
Human|ALX1|Alx|PRD             KRRHRTTFTS LQLEELEK-V FQ-KTHYPDV YVREQLALRT ELTEARVQVW FQNRRAKWRK RE
Mouse|Alx1|Alx|PRD             KRRHRTTFTS LQLEELEK-V FQ-KTHYPDV YVREQLALRT ELTEARVQVW FQNRRAKWRK RE
Chicken|ALX1|Alx|PRD           KRRHRTTFTS LQLEELEK-V FQ-KTHYPDV YVREQLALRT ELTEARVQVW FQNRRAKWRK RE
Frog|alx1|Alx|PRD              KRRHRTTFTS LQLEELEK-V FQ-KTHYPDV YVREQLALRT ELTEARVQVW FQNRRAKWRK RE
Human|ALX3|Alx|PRD             KRRNRTTFST FQLEELEK-V FQ-KTHYPDV YAREQLALRT DLTEARVQVW FQNRRAKWRK RE
Mouse|Alx3|Alx|PRD             KRRNRTTFST FQLEELEK-V FQ-KTHYPDV YAREQLALRT DLTEARVQVW FQNRRAKWRK RE
Human|ALX4|Alx|PRD             KRRNRTTFTS YQLEELEK-V FQ-KTHYPDV YAREQLAMRT DLTEARVQVW FQNRRAKWRK RE
Mouse|Alx4|Alx|PRD             KRRNRTTFTS YQLEELEK-V FQ-KTHYPDV YAREQLAMRT DLTEARVQVW FQNRRAKWRK RE
Chicken|ALX4|Alx|PRD           KRRNRTTFTS YQLEELEK-V FQ-KTHYPDV YAREQLAMRT DLTEARVQVW FQNRRAKWRK RE
Frog|alx4|Alx|PRD              KRRNRTTFTS YQLEELEK-V FQ-KTHYPDV YAREQLAMRT DLTEARVQVW FQNRRAKWRK RE
Human|ARGFX|Argfx|PRD          RHKERTSFTH QQYEELEA-L FS-QTMFPDR NLQEKLALRL DLPESTVKVW FRNRRFKLKK QQ
Human|ARX|Arx|PRD              QRRYRTTFTS YQLEELER-A FQ-KTHYPDV FTREELAMRL DLTEARVQVW FQNRRAKWRK RE
Mouse|Arx|Arx|PRD              QRRYRTTFTS YQLEELER-A FQ-KTHYPDV FTREELAMRL DLTEARVQVW FQNRRAKWRK RE
Chicken|ARX|Arx|PRD            QRRYRTTFST FQLEELER-A FR-KSHYPDV FTREELALRL ELTEARVQVW FQNRRAKWRK RE
Frog|arx|Arx|PRD               QRRYRTTFTS YQLEELER-A FQ-KTHYPDV FTREELAMRL DLTEARVQVW FQNRRAKWRK RE
Frog|arx-l|Arx|PRD             QRRYRTTFSN LQLEELEM-A FR-KSHYPDV FTREDLAMRL DLTEARVQVW FQNRRAKWRK RE
Human|CRX|Otx|PRD              QRRERTTFTR SQLEELEA-L FA-KTQYPDV YAREEVALKI NLPESRVQVW FKNRRAKCRQ QR
Mouse|Crx|Otx|PRD              QRRERTTFTR SQLEELEA-L FA-KTQYPDV YAREEVALKI NLPESRVQVW FKNRRAKCRQ QR
Frog|crx|Otx|PRD               QRRERTTFTR AQLDILEA-L FA-KTRYPDI FMREEVALKI NLPESRVQVW FKNRRAKCRQ QQ
Human|DMBX1|Dmbx|PRD           QRRSRTAFTA QQLEALEK-T FQ-KTHYPDV VMRERLAMCT NLPEARVQVW FKNRRAKFRK KQ
Mouse|Dmbx1|Dmbx|PRD           QRRSRTAFTA QQLEALEK-T FQ-KTHYPDV VMRERLAMCT NLPEARVQVW FKNRRAKFRK KQ
Chicken|DMBX1|Dmbx|PRD         QRRSRTAFTA QQLEALEK-T FQ-KTHYPDV VMRERLAMCT NLPEARVQVW FKNRRAKFRK KQ
Frog|dmbx1|Dmbx|PRD            QRRSRTAFTA QQLEALEK-T FQ-KTHYPDV VMRERLAMCT NLPEARVQVW FKNRRAKFRK KQ
Human|DPRX|Dprx|PRD            SHRKRTMFTK KQLEDLNI-L FN-ENPYPNP SLQKEMASKI DIHPTVLQVW FKNHRAKLKK AK
Human|DRGX|Drgx|PRD            QRRNRTTFTL QQLEALEA-V FA-QTHYPDV FTREELAMKI NLTEARVQVW FQNRRAKWRK TE
Chicken|DRGX|Drgx|PRD          QRRNRTTFTL QQLEALEA-V FA-QTHYPDV FTREELAMKI NLTEARVQVW FQNRRAKWRK TE
Frog|drgx|Drgx|PRD             QRRNRTTFTL QQLEALEA-V FA-QTHYPDV FTREELAMKI NLTEARVQVW FQNRRAKWRK TE
Mouse|Dux|HD1|Dux|PRD          RRRRKMVWQA WQEQALLS-T FK-EKRYLSF KERKELAKGM GVSDCRIRVW FQNRRNRSGE EG
Mouse|Dux|HD2|Dux|PRD          GRRPRTRLTS LQRRILGQ-A FE-RNPRPGF ATREELARDT GLPEDTIHIW FQNRRARWRH RR
Human|DUX4|HD1|Dux|PRD         GRRRRLVWTP SQSEALRA-C FE-RNPYPGI ATRERLAQAI GIPEPRVQIW FQNERSRQLR QH
Human|DUX4|HD2|Dux|PRD         GRRKRTAVTG SQTALLLR-A FE-KDRFPGI AAREELARET GLPESRIQIW FQNRRARHPG QG
Human|Dux4l|HD1|Dux|PRD        --QQRIVLNS SQKDALQA-L FK-QNSYPGI PTREQLAKEI GSLESRIQIW FQNQ------ --
Human|Dux4l|HD2|Dux|PRD        ------AITK PQTGVLLQ-A FQ-RNRFPGT AIKEELAKQT GIPESRI--- ---------- --
Human|DUX4L10|HD1|Dux|PRD      GRRRRLVWTP SQSEALRA-C FE-RNPYPGI ATRERLAQAI GIPEPRVQIW FQNERSRQLR QH
Human|DUX4L10|HD2|Dux|PRD      GRRKRTAVTG SQTALLLR-A FE-KDRFPGI AAREELARET GLPESRIQIW FQNRRARHPG QG
Human|DUX4L11|HD1|Dux|PRD      GRRRRLVWTP SQSEALRA-C FE-RNPYPGI ATRERLAQAI GIPEPRVQIW FQNERSRQLR QH
Human|DUX4L11|HD2|Dux|PRD      GRRKRTAVTG SQTALLLR-A FE-KDRFPGI AAREELARET GLPESRIQIW FQNRRARHPG QG
Human|DUX4L12|HD1|Dux|PRD      GRRRRLVWTP SQSEALRA-C FE-RNPYPGI ATRERLAQAI GIPEPRVQIW FQNERSRQLR QH
Human|DUX4L12|HD2|Dux|PRD      GRRKRTAVTG FQTALLLR-A FE-KDRFPGI AAREELARET GLPESRIQIW FQNRRARHPG QG
Human|DUX4L13|HD1|Dux|PRD      GRRRRLVWTP SQSEALRA-C FE-RNPYPGI ATRERLAQAI GIPEPRVQIW FQNERSRQLR QH
Human|DUX4L13|HD2|Dux|PRD      GRRKRTAVTG SQTALLLR-A FE-KDRFPGI AAREELARET GLPESRIQIW FQNRRARHPG QG
Human|DUX4L14|HD1|Dux|PRD      GRRRRLVWTP SQSEALRA-C FE-RNPYPGI ATRERLAQAI GIPEPRVQIW FQNERSRQLR QH
Human|DUX4L14|HD2|Dux|PRD      GRRKRTAVTG SQTALLLR-A FE-KDRFPGI AAREELARET GLPESRIQIW FQNRRARHPG QG
Human|DUX4L15|HD1|Dux|PRD      GRRKRTAVTG SQTALLLR-A FE-KDRFPGI AAREELARET GLPESRIQIW FQNRRARHPG QG
Human|DUX4L15|HD2|Dux|PRD      GRRRRLVWTP SQSEALRA-C FE-RNPYPGI ATRERLAQAI GIPEPRVQIW FQNERSRQLR QH
Human|DUX4L2|HD1|Dux|PRD       GRRRRLVWTP SQSEALRA-C FE-RNPYPGI ATRERLAQAI GIPEPRVQIW FQNERSRQLR QH
Human|DUX4L2|HD2|Dux|PRD       GRRKRTAVTG SQTALLLR-A FE-KDRFPGI AAREELARET GLPESRIQIW FQNRRARHPG QG
Human|DUX4L3|HD1|Dux|PRD       GRRRRLVWTP SQSEALRA-C FE-RNPYPGI ATRERLAQAI GIPEPRVQIW FQNERSRQLR QH
Human|DUX4L3|HD2|Dux|PRD       GRRKRTAVTG SQTALLLR-A FE-KDRFPGI AAREELARET GLPESRIQIW FQNRRARHPG QG
Human|DUX4L4|HD1|Dux|PRD       GRRRRLVWTP SQSEALRA-C FE-RNPYPGI ATRERLAQAI GIPEPRVQIW FQNERSRQLR QH
Human|DUX4L4|HD2|Dux|PRD       GRRKRTAVTG SQTALLLR-A FE-KDRFPGI AAREELARET GLPESRIQIW FQNRRARHPG QG
Human|DUX4L5|HD1|Dux|PRD       GRRRRLVWTP SQSEALRA-C FE-RNPYPGI ATRERLAQAI GIPEPRVQIW FQNERSRQLR QH
Human|DUX4L5|HD2|Dux|PRD       GRRKRTAVTG SQTALLLR-A FE-KDRFPGI AAREELARET GLPESRIQIW FQNRRARHPG QG
Human|DUX4L6|HD1|Dux|PRD       GRRRRLVWTP SQSEALRA-C FE-RNPYPGI ATRERLAQAI GIPEPRVQIW FQNERSRQLR QH
Human|DUX4L6|HD2|Dux|PRD       GRRKRTAVTG SQTALLLR-A FE-KDRFPGI AAREELARET GLPESRIQIW FQNRRARHPG QG
Human|DUX4L7|HD1|Dux|PRD       GRRRRLVWTP SQSEALRA-C FE-RNPYPGI ATRERLAQAI GIPEPRVQIW FQNERSRQLR QH
Human|DUX4L7|HD2|Dux|PRD       GRRKRTAVTG SQTALLLR-A FE-KDRFPGI AAREELARET GLPESRIQIW FQNRRARHPG QG
Human|DUX4L8|HD1|Dux|PRD       GRRRRLVWTP SQSEALRA-C FE-RNPYPGI ATRERLAQAI GIPEPRVQIW FQNERSRQLR QH
Human|DUX4L8|HD2|Dux|PRD       GRRKRTAVTG SQTALLLR-A FE-KDRFPGI AAREELARET GLPESRIQIW FQNRRARHPG QG
Human|DUX4L9|HD1|Dux|PRD       GRRRRLVWTP SQSEALRA-C FE-RNPYPGI ATRERLAQAI GIPEPRVQIW FQNERSRQLR QH
Human|DUX4L9|HD2|Dux|PRD       GRRKRTAVTG SQTALLLR-A FE-KDRFPGI AAREELARET GLPESRIQIW FQNRRARHPG QG
Human|DUXA|HD1|Dux|PRD         HRRCRTKFTE EQLKILIN-T FN-QKPYPGY ATKQKLALEI NTEESRIQIW FQNRRARHGF QK
Human|DUXA|HD2|Dux|PRD         ARRCRTTYSA SQLHTLIK-A FM-KNPYPGI DSREELAKEI GVPESRVQIW FQNRRSRLLL QR
Human|DUXB|HD1|Dux|PRD         FWRNRIQYNQ SQKDILQS-W FQ-HDPFPDK AAREQLAKEI GVPESNIQVW FKNYRVKQRK LD
Human|DUXB|HD2|Dux|PRD         ARQKQTFITW TQKNRLVQ-A FE-RNPFPDI ATRKKLAEQT GLQESRIQMW FQKQRSLYLK KS
Mouse|Duxbl|HD1|Dux|PRD        ARRRRIILTQ SQKDTLRV-W FE-KNPNPDL ATRGHLAKEL GISESQIMTW FQKHRKIRKQ AE
Mouse|Duxbl|HD2|Dux|PRD        ARRSRTHFTK FQTDILIE-A FE-KNRFPGI VTREKLAQQT GIPESRIHIW FQNRRARHPD PG
Human|ESX1|Esx|PRD             KRRRRTAFTQ FQLQELEN-F FD-ESQYPDV VARERLAARL NLTEDRVQVW FQNRRAKWKR NQ
Mouse|Esx1|Esx|PRD             PRRYRICFTP IQLQELEA-F FQ-RVQYPDL FARVELARRL GLPEPRVQVW FQNRRAKWRR LR
Mouse|Gm10391|HD1|Dux|PRD      ARRRRIILTQ SQKDTLRV-W FE-KNPNPDL ATRGHLAKEL GISESQIMTW FQKHRKIRKQ AE
Mouse|Gm10391|HD2|Dux|PRD      ARRSRTHFTK FQTDILIE-A FE-KNRFPGI VTREKLAQQT GIPESRIHIW FQNRRARHPD PG
Mouse|Gm10394|HD1|Dux|PRD      ARRRRIILTQ SQKDTLRV-W FE-KNPNPDL ATRGHLAKEL GISESQIMTW FQKHRKIRKQ AE
Mouse|Gm10394|HD2|Dux|PRD      ARRSRTHFTK FQTDILIE-A FE-KNRFPGI VTREKLAQQT GIPESRIHIW FQNRRARHPD PG
Mouse|Gm10678|Obox|PRD         CRKERTVYSK EQKCLLQE-H FH-QCQNPDL EQRKALALLI GVTEYKIQTW FKNRRAKECR KS
Mouse|Gm10679|Obox|PRD         CRKEHTVYSK EQKCLLQE-H FH-QCQNPDL EQRKALALLI GVTEYKIQTW FKNRRAKECR KS
Mouse|Gm14543|Rhox|PRD         HRPLRDRFTE PQLQELEQ-V FQ-RNHYLRA EEGKQLARGM GVTEAKLQRW FKKRRVQFRR EH
Mouse|Gm14544|Rhox|PRD         YCRKQYKFTP EQLLELDR-I FE-ETQCPDT LQRKEIAKLM NVEECTVKIW FCNQRAKLRK NQ
Mouse|Gm3977|Obox|PRD          CRKERTVYSK EQKCLLQE-H FH-QCQNPDL EQRKALALLI GVTEYKIQTW FKNRRAKECR KS
Mouse|Gm3980|Obox|PRD          CRKERTVYSK EQKCLLQE-H FH-QCQNPDL EQRKALALLI GVTEYKIQTW FKNRRAKECR KS
Mouse|Gm3981|Obox|PRD          CRKERTVYSK EQKCLLQE-H FH-QCQNPDL EQRKALALLI GVTEYKIQTW FKNRRAKECR KS
Mouse|Gm3984|Obox|PRD          CRKERTVYSK EQKCLLQE-H FH-QCQNPDL EQRKALALLI GVTEYKIQTW FKNRRAKECR KS
Mouse|Gm3987|Obox|PRD          CRKERTVYSK EQKCLLQE-H FH-QWQNPDL EQRKALALLI GVTEYKIQTW FKNRRAKECR KS
Mouse|Gm3989|Obox|PRD          CRKERTVYSK EQKCLLQE-H FH-QCQNPDL EQRKALALLI GVTEYKIQTW FKNRRAKECR KS
Mouse|Gm3994|Obox|PRD          CRKERTVYSK EQKCLLQE-H FH-QCQNPDL EQRKALALLI GVTEYKIQTW FKNRRAKECR KS
Mouse|Gm3996|Obox|PRD          CRKERTVYSK EQKCLLQE-H FH-QCQNPDL EQRKALALLI GVTEYKIQTW FKNRRAKECR KS
Mouse|Gm3998|Obox|PRD          CRKERTVYSK EQKCLLQE-H FH-QCQNPDL EQRKALALLI GVTEYKIQTW FKNRRAKECR KS
Mouse|Gm4001|Obox|PRD          CRKERTVYSK EQKCLLQE-H FH-QCQNPDL EQRKALALLI GVTEYKIQTW FKNRRAKECR KS
Mouse|Gm4003|Obox|PRD          CRKERTVYSK EQKCLLQE-H FH-QCQNPDL EQRKALALLI GVTEYKIQTW FKNRRAKECR KS
Mouse|Gm4023|Obox|PRD          CRKERTVYSK EQKCLLQE-H FH-QCQNPDL EHRKALALLI GVTEYKIQTW FKNRRAKECR KS
Mouse|Gm4745|Obox|PRD          FRKERIVYSK EQQRLLQK-H FD-ECQYPKE KKIVELAVLI GVTKMEIKKW FKNNRAKYRQ MN
Mouse|Gm4897|Obox|PRD          CRKERTVYSK EQKCLLQE-H FH-QCQNPDL EQRKALALLI GVTEYKIQTW FKNRRAKECR KS
```

```
Mouse|Gm4981|Dux|PRD                 SRRPHTGLTL SQRRILAQ-A FE-RNPRPGC ATREELALET GLPEDMIHTW LKNKRARRHR RG
Mouse|Gm5889|Obox|PRD                FRKERTVYSK EQQCLLQK-H FD-ECQYPNE KKIVELALSV GVTKREIKIW FKNNGAKYRR MK
Mouse|Gm6310|Rhox|PRD                DLCLRDGFTE PQLQELEQ-V FQ-RNHYLRA EEGKQLARGM GVTEAKLQRW FKKRRVQFRR EQ
Mouse|Gm7590|Rhox|PRD                HRPLRDRFTE PQLQELEQ-V FQ-RNHCLRA EEGKQLARGM GVTEAKLQRW FKKRRVQFRR EH
Mouse|Gm8040|Obox|PRD                FRKERTVYSK EQQCLLQK-H FD-ECQYPNE KKIVELALSV GVTKREIKIW FKNNRAKYRR MK
Mouse|Gm8053|Obox|PRD                FRKERTVYSK EQQCLLQK-H FD-ECQYPNE KKIVELALSV GVTKREIKIW FKNNRAKYRR MK
Mouse|Gm8072|Obox|PRD                FRKEHTVYSK EQQGLLQK-H FD-ECQYPNK KKIMELALSV GVTKREIKIW FKNNRAKYRQ MK
Human|GSC|Gsc|PRD                    KRRHRTIFTD EQLEALEN-L FQ-ETKYPDV GTREQLARKV HLREEKVEVW FKNRRAKWRR QK
Mouse|Gsc|Gsc|PRD                    KRRHRTIFTD EQLEALEN-L FQ-ETKYPDV GTREQLARKV HLREEKVEVW FKNRRAKWRR QK
Chicken|GSC|Gsc|PRD                  KRRHRTIFTD EQLEALEN-L FQ-ETKYPDV GTREQLARKV HLREEKVEVW FKNRRAKWRR QK
Frog|gsc|Gsc|PRD                     KRRHRTIFTD EQLEALEN-L FQ-ETKYPDV GTREQLARRV HLREEKVEVW FKNRRAKWRR QK
Human|GSC2|Gsc|PRD                   TRRHRTIFSE EQLQALEA-L FV-QNQYPDV STRERLAGRI RLREERVEVW FKNRRAKWRH QK
Mouse|Gsc2|Gsc|PRD                   TRRHRTIFSE EQLQALEA-L FV-QNQYPDV GTRERLAVRI RLREERVEVW FKNRRAKWRH QK
Chicken|GSC2|Gsc|PRD                 TRRHRTIFTE EQLQALET-L FH-QNQYPDV ITREHLANRI HLKEERVEVW FKNRRAKWRH QK
Human|HESX1|Hesx|PRD                 GRRPRTAFTQ NQIEVLEN-V FR-VNCYPGI DIREDLAQKL NLEEDRIQIW FQNRRAKLKR SH
Mouse|Hesx1|Hesx|PRD                 GRRPRTAFTQ NQVEVLEN-V FR-VNCYPGI DIREDLAQKL NLEEDRIQIW FQNRRAKMKR SR
Chicken|HESX1|Hesx|PRD               GRRPRTAFTR NQIEVLEN-V FK-MNSYPGI DIREELARKL DLEEDRIQIW FQNRRAKLKR SH
Frog|hesx1|Hesx|PRD                  GRRPRTAFTR SQIEILEN-V FR-VNSYPGI DIREELAGKL ALDEDRIQIW FQNRRAKLKR SH
Human|HOPX|Hopx|PRD                  SAETASGPTE DQVEILEY-N FNKVDKHPDS TTLCLIAAEA GLSEEETQKW FKQRLAKWRR SE
Mouse|Hopx|Hopx|PRD                  -AQTASGPTE DQVEILEY-N FNKVNKHPDP TTLCLIAAEA GLTEEQTQKW FKQRLAEWRR SE
Chicken|HOPX|Hopx|PRD                ATEKSVTPTE EQLEILEY-N FCKVNKHPDP TTLCLIAAET GLSEEQTLKW FKQRLAEWRK SE
Frog|hopx|Hopx|PRD                   -GSDSTGLTN QQIEVLEY-N FNNVSKQPHN TSIMLIAAET GLTEEETKKW FKERLAKWRE SE
Human|ISX|Isx|PRD                    KRRVRTTFTT EQLHELEK-I FH-FTHYPDV HIRSQLAARI NLPEARVQIW FQNQRAKWRK QE
Mouse|Isx|Isx|PRD                    KRRVRTTFTT EQLQELEK-L FH-FTHYPDI HVRSQLASRI NLPEARVQIW FQNQRAKWRK QE
Chicken|ISX|Isx|PRD                  KRRIRTTFTA EQLQELEK-I FQ-VTHYPDV HIRNQLAAKI NLPEARVQIW FQNQRAKWRK HE
Frog|isx|Isx|PRD                     KRRIRTTFTL AQLQELEQ-I FQ-VTHYPDV QTRDQLAAKI QLPEARVQIW FQNRRAKWRK YE
Mouse|LOC100504180|HD1|Dux|PRD       RRRRKMVWQA WQEQALLS-T FK-EKRYLSF KERKELAKGM GVSDCRIRVW FQNRRNRSGE EG
Mouse|LOC100504180|HD2|Dux|PRD       GRRPRTRLTS LQRRILGQ-A FE-RNPRPGF ATREELARDT GLPEDTIHIW FQNRRARWRH RR
Human|LOC100506764|HD1|Dux|PRD       GRRRRLVWTP SQSEVLRA-C FE-RNPYPGI ATRERLAQAI GIPEPRVQIW FQNERSRQLR QH
Human|LOC100506764|HD2|Dux|PRD       GRRKRTAVTG SQTALLLR-A FE-KDRFPGI AAREELARET GLPESRIQIW FQNRRARHPG QG
Chicken|LOC419032|Nobox|PRD          -KKTRTFYSA EQLEELEK-V FQ-EDRYPDN EKRREIAAVI GVTPQRIMVW FQNRRAKWRK T-
Chicken|LOC769839|Mix|PRD            -RRKRTSFSK AQLELLVR-T FE-KQPYPGI ALREQLSGLT DIPESRIQV- ---------- --
Chicken|LOC771564|Otp|PRD            QKRHRTRFTP AQLNELER-R FA-KTHYPDI FMREELALRI GLTESRVQV- ---------- --
Human|MIXL1|Mix|PRD                  QRRKRTSFSA EQLQLLEL-V FR-RTRYPDI HLRERLAALT LLPESRIQVW FQNRRAKSRR QS
Mouse|Mixl1|Mix|PRD                  QRRKRTSFSS EQLQLLEL-V FR-QTMYPDI HLRERLAALT LLPESRIQVW FQNRRAKSRR QS
Chicken|MIXL1|Mix|PRD                -RRKRTSFTA AQLETLEL-V FQ-DTMYPDI YLRERLADAT QIPESRIQVW FQNRRAK--- --
Human|NOBOX|Nobox|PRD                RKKTRTLYRS DQLEELEK-I FQ-EDHYPDS DKRREIAQTV GVTPQRIMVW FQNRRAKWRK ME
Mouse|Nobox|Nobox|PRD                RKKTRTLYRS DQLEELER-I FQ-EDHYPDS DKRHEISQMV GVTPQRIMVW FQNRRAKWRK VE
Frog|nobox-1|Nobox|PRD               RKKSRTLYSM DQLQELER-L FA-EDHYPDS EKRREIAEII GVTPQRIMVW FQNRRAKWRK VE
Mouse|Obox1|Obox|PRD                 FRKERTVYTK EQQGLLQK-H FD-ECQYPNK KKIVELALSV GVTKREIKIW FKNNRAKYRR MN
Mouse|Obox2|Obox|PRD                 FRKERTVYTK EEQGLLQK-H FD-ECQYPNK KKIVELALSV GVTKREIKIW FKNNRAKYRQ MN
Mouse|Obox3|Obox|PRD                 FRKERTVYSK EQQCLLQK-H FD-EFQYPNE KKIVELALSV GVTKREIKIW FKNNRAKYRR MK
Mouse|Obox5|Obox|PRD                 FRKERTVYTK EQQRLLQK-H FD-ECQYPNE KKIMELAVSV GVTKMEIKIW FKNNRAKYRR MN
Mouse|Obox6|Obox|PRD                 HRKIRTVYTE EQKCVLKK-H FH-KCTYPSR EQRMALAVLV GVTANEIQIW FKNHRAKSKR ES
Human|OTP|Otp|PRD                    QKRHRTRFTP AQLNELER-S FA-KTHYPDI FMREELALRI GLTESRVQVW FQNRRAKWKK RK
Mouse|Otp|Otp|PRD                    QKRHRTRFTP AQLNELER-S FA-KTHYPDI FMREELALRI GLTESRVQVW FQNRRAKWKK RK
Frog|otp-l|Otp|PRD                   -KRHRTRFTP AQLNELER-S FA-KTHYPDI FMREELALRI GLTESRVQV- ---------- --
Frog|otpa|Otp|PRD                    QKRHRTRFTP AQLNELER-S FA-KTHYPDI FMREELALRI GLTESRVQV- ---------- --
Frog|otpb|Otp|PRD                    QKRHRTRFTP AQLNELER-S FA-KTHYPDI FMREELALRI GLTESRVQVW FQNRRAKWKK RK
Human|OTX1|Otx|PRD                   QRRERTTFTR SQLDVLEA-L FA-KTRYPDI FMREEVALKI NLPESRVQVW FKNRRAKCRQ QQ
Mouse|Otx1|Otx|PRD                   QRRERTTFTR SQLDVLEA-L FA-KTRYPDI FMREEVALKI NLPESRVQVW FKNRRAKCRQ QQ
Frog|otx1|Otx|PRD                    QRRERTTFTR SQLDVLEA-L FA-KTRYPDI FMREEVALKI NLPESRVQVW FKNRRAKCRQ QQ
Human|OTX2|Otx|PRD                   QRRERTTFTR AQLDVLEA-L FA-KTRYPDI FMREEVALKI NLPESRVQVW FKNRRAKCRQ QQ
Mouse|Otx2|Otx|PRD                   QRRERTTFTR AQLDVLEA-L FA-KTRYPDI FMREEVALKI NLPESRVQVW FKNRRAKCRQ QQ
Frog|otx2|Otx|PRD                    QRRERTTFTR AQLDVLEA-L FA-KTRYPDI FMREEVALKI NLPESRVQVW FKNRRAKCRQ QQ
Human|PAX2|Pax2/5/8|PRD              KHLRADTFTQ QQLEALDR-V FE-RPSYPDV FQASE----- ---------- ---------- --
Frog|pax2|Pax2/5/8|PRD               KHLRADNFTQ QQLEALDR-V FE-RPSYPDV FQTAE----- ---------- ---------- --
Mouse|Pax2|Pax2/5/8|PRD              KHLRADTFTQ QQLEALDR-V FE-RPSYPDV FQASE----- ---------- ---------- --
Human|PAX3|Pax3/7|PRD                QRRSRTTFTA EQLEELER-A FE-RTHYPDI YTREELAQRA KLTEARVQVW FSNRRARWRK QA
Mouse|Pax3|Pax3/7|PRD                QRRSRTTFTA EQLEELER-A FE-RTHYPDI YTREELAQRA KLTEARVQVW FSNRRARWRK QA
Chicken|PAX3|Pax3/7|PRD              QRRSRTTFTA EQLEELER-A FE-RTHYPDI YTREELAQRA KLTEARVQVW FSNRRARWRK QA
Human|PAX4|Pax4/6|PRD                GHRNRTIFSP SQAEALEK-E FQ-RGQYPDS VARGKLATAT SLPEDTVRVW FSNRRAKWRR QE
Mouse|Pax4|Pax4/6|PRD                SHRNRTIFSP GQAEALEK-E FQ-RGQYPDS VARGKLAAAT SLPEDTVRVW FSNRRAKWRR QE
Frog|pax4l|Pax4/6|PRD                --RNRTVFST AQAEVLEKEE FM-RVQYPDI SRREKLATDT ALSEITIRVW FSNRRAKWRR E-
Human|PAX5|Pax2/5/8|PRD              KQMRGDLFTQ QQLEVLDR-V FE-RQHYSDI FTTTE----- ---------- ---------- --
Mouse|Pax5|Pax2/5/8|PRD              KQMRGDLFTQ QQLEVLDR-V FE-RQHYSDI FTTTE----- ---------- ---------- --
Human|PAX6|Pax4/6|PRD                LQRNRTSFTQ EQIEALEK-E FE-RTHYPDV FARERLAAKI DLPEARIQVW FSNRRAKWRR EE
Mouse|Pax6|Pax4/6|PRD                LQRNRTSFTQ EQIEALEK-E FE-RTHYPDV FARERLAAKI DLPEARIQVW FSNRRAKWRR EE
Chicken|PAX6|Pax4/6|PRD              LQRNRTSFTQ EQIEALEK-E FE-RTHYPDV FARERLAAKI DLPEARIQVW FSNRRAKWRR EE
Frog|pax6|Pax4/6|PRD                 LQRNRTSFTQ EQIEALEK-E FE-RTHYPDV FARERLAAKI DLPEARIQVW FSNRRAKWRR EE
Frog|pax6l|Pax4/6|PRD                LQRNRTSFTQ EQIEALEK-E FE-RTHYPDV FARERLAAKI DLPEARIQVW FSNRRAKWRR EE
Human|PAX7|Pax3/7|PRD                QRRSRTTFTA EQLEELEK-A FE-RTHYPDI YTREELAQRT KLTEARVQVW FSNRRARWRK QA
Mouse|Pax7|Pax3/7|PRD                QRRSRTTFTA EQLEELEK-A FE-RTHYPDI YTREELAQRT KLTEARVQVW FSNRRARWRK QA
Chicken|PAX7|Pax3/7|PRD              QRRSRTTFTA EQLEELEK-A FE-RTHYPDI YTREELAQRT KLTEARVQVW FSNRRARWRK QA
Frog|pax7|Pax3/7|PRD                 QRRSRTTFTA EQLEELEK-A FE-RTHYPDI YTREELAQRT KLTEARVQVW FSNRRARWRK QA
Human|PAX8|Pax2/5/8|PRD              KHLRTDAFSQ HHLEPLEC-P FE-RQHYPEA YASPS----- ---------- ---------- --
Mouse|Pax8|Pax2/5/8|PRD              KHLRTDTFSQ HHLEALEC-P FE-RQHYPEA YASPS----- ---------- ---------- --
Human|PHOX2A|Phox|PRD                QRRIRTTFTS AQLKELER-V FA-ETHYPDI YTREELALKI DLTEARVQVW FQNRRAKFRK QE
Mouse|Phox2a|Phox|PRD                QRRIRTTFTS AQLKELER-V FA-ETHYPDI YTREELALKI DLTEARVQVW FQNRRAKFRK QE
Frog|phox2a|Phox|PRD                 QRRIRTTFTS SQLKELER-V FA-ETHYPDI YTREELALKI DLTEARVQVW FQNRRAKFRK QE
Human|PHOX2B|Phox|PRD                QRRIRTTFTS AQLKELER-V FA-ETHYPDI YTREELALKI DLTEARVQVW FQNRRAKFRK QE
Mouse|Phox2b|Phox|PRD                QRRIRTTFTS AQLKELER-V FA-ETHYPDI YTREELALKI DLTEARVQVW FQNRRAKFRK QE
Chicken|PHOX2B|Phox|PRD              QRRIRTTFTS AQLKELER-V FA-ETHYPDI YTREELALKI DLTEARVQVW FQN------- --
Frog|phox2b|Phox|PRD                 QRRIRTTFTS AQLKELER-V FA-ETHYPDI YTREELALKI DLTEARVQVW FQNRRAKFRK QE
Human|PITX1|Pitx|PRD                 QRRQRTHFTS QQLQELEA-T FQ-RNRYPDM SMREEIAVWT NLTEPRVRVW FKNRRAKWRK RE
Mouse|Pitx1|Pitx|PRD                 QRRQRTHFTS QQLQELEA-T FQ-RNRYPDM SMREEIAVWT NLTEPRVRVW FKNRRAKWRK RE
Chicken|PITX1|Pitx|PRD               QRRQRTHFTS QQLQELEA-T FQ-RNRYPDM SMREEIAVWT NLTEPRVRVW FKNRRAKWRK RE
Frog|pitx1|Pitx|PRD                  QRRQRTHFTS QQLQELEA-T FQ-RNRYPDM SMREEIAVWT NLTEARVRVW FKNRRAKWRK RE
Human|PITX2|Pitx|PRD                 QRRQRTHFTS QQLQELEA-T FQ-RNRYPDM STREEIAVWT NLTEARVRVW FKNRRAKWRK RE
Mouse|Pitx2|Pitx|PRD                 QRRQRTHFTS QQLQELEA-T FQ-RNRYPDM STREEIAVWT NLTEARVRVW FKNRRAKWRK RE
Chicken|PITX2|Pitx|PRD               QRRQRTHFTS QQLQELEA-T FQ-RNRYPDM STREEIAVWT NLTEARVRVW FKNRRAKWRK RE
Frog|pitx2|Pitx|PRD                  QRRQRTHFTS QQLQELEA-T FQ-RNRYPDM STREEIAVWT NLTEARVRVW FKNRRAKWRK RE
Frog|pitx2b|Pitx|PRD                 QRRQRTHFTS QQLQELEA-T FQ-RNRYPDM STREEIAVWT NLTEARVRVW FKNRRAKWRK RE
Human|PITX3|Pitx|PRD                 QRRQRTHFTS QQLQELEA-T FQ-RNRYPDM STREEIAVWT NLTEARVRVW FKNRRAKWRK RE
Mouse|Pitx3|Pitx|PRD                 QRRQRTHFTS QQLQELEA-T FQ-RNRYPDM STREEIAVWT NLTEARVRVW FKNRRAKWRK RE
Chicken|PITX3|Pitx|PRD               QRRQRTHFTS QQLQELEA-T FQ-RNRYPDM STREEIAVWT NLTEARVRVW FKNRRAKWRK RE
Human|PROP1|Prop|PRD                 RRRHRTTFSP VQLEQLES-A FG-RNQYPDI WARESLARDT GLSEARIQVW FQNRRAKQRK QE
Mouse|Prop1|Prop|PRD                 RRRHRTTFNP AQLEQLES-A FG-RNQYPDI WAREGLAQDT GLSEARIQVW FQNRRAKQRK QE
Human|PRRX1|Prrx|PRD                 QRRNRTTFNS SQLQALER-V FE-RTHYPDA FVREDLARRV NLTEARVQVW FQNRRAKFRR NE
```

```
Mouse|Prrx1|Prrx|PRD          QRRNRTTFNS SQLQALER-V FE-RTHYPDA FVREDLARRV NLTEARVQVW FQNRRAKFRR NE
Chicken|PRRX1|Prrx|PRD        QRRNRTTFNS SQLQALER-V FE-RTHYPDA FVREDLARRV NLTEARVQVW FQNRRAKFRR NE
Frog|prrx1b|Prrx|PRD          QRRNRTTFNS SQLQALER-V FE-RTHYPDA FVREDLARRV NLTEARVQVW FQNRRAKFRR NE
Human|PRRX2|Prrx|PRD          QRRNRTTFNS SQLQALER-V FE-RTHYPDA FVREELARRV NLSEARVQVW FQNRRAKFRR NE
Mouse|Prrx2|Prrx|PRD          QRRNRTTFNS SQLQALER-V FE-RTHYPDA FVREELARRV NLSEARVQVW FQNRRAKFRR NE
Chicken|PRRX2|Prrx|PRD        QRRNRTTFNS SQLQALER-V FE-RTHYPDA FVREELARRV NLSEARVQVW FQNRRAKFRR NE
Frog|prrx2|Prrx|PRD           QRRNRTTFNS SQLQALER-V FE-RTHYPDA FVREDLARRV SLSEARVQVW FQNRRAKFRR NE
Mouse|Prrxl1|Drgx|PRD         QRRNRTTFTL QQLEALEA-V FA-QTHYPDV FTREELAMKI NLTEARVQVW FQNRRAKWRK TE
Human|RAX|Rax|PRD             HRRNRTTFTT YQLHELER-A FE-KSHYPDV YSREELAGKV NLPEVRVQVW FQNRRAKWRR QE
Mouse|Rax|Rax|PRD             HRRNRTTFTT YQLHELER-A FE-KSHYPDV YSREELAGKV NLPEVRVQVW FQNRRAKWRR QE
Frog|rax|Rax|PRD              HRRNRTTFTT YQLHELER-A FE-KSHYPDV YSREELAMKV NLPEVRVQVW FQNRRAKWRR QE
Human|RAX2|Rax|PRD            HRRNRTTFTT YQLHQLER-A FE-ASHYPDV YSREELAAKV HLPEVRVQVW FQNRRAKWRR QE
Frog|rax2                     HRRNRTTFTT YQLHELER-A FE-RSHYPDV YSREELAMKV SLPEVRVQVW FQNRRAKWRR QE
Mouse|Rhox1|Rhox|PRD          KCGLSNRFSR WQLQQLEL-L FQ-RTQYISA QDRKRLAVCL CVCEAKVQNW FQRRRAEYRK YH
Mouse|Rhox10|Rhox|PRD         RRSNSKKYTN AQMCELEK-A FQ-ETQYPDA HQRKALAKLI DVDECKVKAW FKYKRAKYRR KQ
Mouse|Rhox11|Rhox|PRD         IPRKAYRFTP GQLWELQA-V FV-ENQYPDA LKRKELAGLL NVDEQKIKDW FNNKRAKYRK IQ
Mouse|Rhox12|Rhox|PRD         RPRIQLGFTP RQLNELED-F FE-KTKYPDA LTRKNLAKHL YLAESKVQRW FKKRRAHYRK EQ
Mouse|Rhox13|Rhox|PRD         RRGPPFHFAQ WQVEEMES-L FE-ETQYPDL LTRGELARTL NVPEVKVKVW FTNRRAKQRK IE
Mouse|Rhox2a|Rhox|PRD         RHGWQQSFNV LQLQELES-I FQ-CNHYIST KEANRLARSM GVSEATVQEW FLKRREKYRS YK
Mouse|Rhox2b|Rhox|PRD         RHGWQQSFNV LQLQELES-I FQ-CNHYIST KEANRLARSM GVSEATVQEW FLKRREKYRS YK
Mouse|Rhox2c|Rhox|PRD         RHGWQQSFNV LQLQELES-I FQ-CNHYIST KEANRLARSM GVSEATVQEW FLKRREKYRS YK
Mouse|Rhox2d|Rhox|PRD         RYSWQQSFNV LQLQELES-I FQ-CNQYIST TEAKRLAKSM GVSEATVQEW FLKRREKYRS YK
Mouse|Rhox2e|Rhox|PRD         RHGWQQSFNV LQLQELES-I FQ-CNHYIST KEANRLARSM GVSEATVQEW FLKRREKYRS YK
Mouse|Rhox2f|Rhox|PRD         RHLWRHSFNV LQLQELES-I FQ-CNHYIST KEANRLARSM GVSEATVQEW FLKRREKYRS YK
Mouse|Rhox2g|Rhox|PRD         LHGWQQSFNV LQLQELES-I FQ-CNHYIST TEAKCLARSM GVSKATVQEW FLKRREKYRS YK
Mouse|Rhox2h|Rhox|PRD         RHLWRHNFNV LQLQELES-I FQ-CNHYIST TEENRLARSM GVSEATVQEW FLKRREKYRS YK
Mouse|Rhox3a|Rhox|PRD         RRRLHHRFTQ WQLDELER-I FR-MNYFLSL EARKQLARWM GVNEAIVKRW FQKRREQYRW YK
Mouse|Rhox3b|Rhox|PRD         RRRLHHRFTQ WQLDELER-I FR-MNYFLSL EARKQLARWM GVNEAIVKRW FQKRREQYRW YK
Mouse|Rhox3c|Rhox|PRD         RRRLHHRFTQ WQLDELER-I FR-MNYFLSL EARKQLARWM GVNEAIVKRW FQKRREQYRW YK
Mouse|Rhox3e|Rhox|PRD         RRRLHHRFTQ WQLDELER-I FR-MNYFLSL EARKQLARWM GVNEAIVKRW FQKRREQYRW YK
Mouse|Rhox3f|Rhox|PRD         RRRLHHRFTQ WQLDELER-I FR-MNYFLSL EARKQLARWM GVNEAIVKRW FQKRREKYRW YK
Mouse|Rhox3g|Rhox|PRD         RRRLHHRFTQ WQLDELER-I FR-MNYFLSL EARKQLARWM GVNEAIVKRW FQKRREQYRW YK
Mouse|Rhox3h|Rhox|PRD         RRRLHHRFTQ WQLDELER-I FR-MNYFLSL EARKQLARWM GVNEAIVKRW FQKRREQYRW YK
Mouse|Rhox4a|Rhox|PRD         QRSLHYNFQW WQLQELER-I FQ-QNHFIRA EERRHLARWI GVSEARVKRW FKKRREHFRR GQ
Mouse|Rhox4b|Rhox|PRD         QRSLHYNFQW WQLQELER-I FQ-QNHFIRA EERRHLARWI GVSEARVMTW FKKRREHFRR GQ
Mouse|Rhox4c|Rhox|PRD         QRSLHYNFQW WQLQELER-I FQ-QNHFIRA EERRHLARWI GVSETRVKRW FKKRREHFRR GQ
Mouse|Rhox4d|Rhox|PRD         QRSLHYNFQW WQLQELER-I FQ-QNHFIRA EERRHLARWI GVSEARVMRW FKKRREHFRR GQ
Mouse|Rhox4e|Rhox|PRD         QRSLHYNFQW WQLQELER-I FQ-QNHFIRA EERRHLARWI GVSETRVKRW FKKRREHFRR GQ
Mouse|Rhox4f|Rhox|PRD         QRSLHYNFQW WQLQELER-I FQ-QNHFIRA EERRHLARWI GVSEARVMTW FKKRREHFRR GQ
Mouse|Rhox4g|Rhox|PRD         QRSLHYNFQW WQLQELER-I FQ-QNHFIRA EERRHLARWI GVSEARVMTW FKKRREHFRR GQ
Mouse|Rhox6|Rhox|PRD          LRYRRTRFTH SQLHDLER-L FQ-ETRYPSL RARRDLARWM GVDECDVQNW FRMRRALFQR NR
Mouse|Rhox7|HD1|Rhox|PRD      ---------- -----MET-M FQ-ETQYPDV LTREVLARSM DGSEAKVQIR FNNRRAKQRA RE
Mouse|Rhox7|HD2|Rhox|PRD      HRPLRDGFTE PQLQELEQ-V FQ-RNHYLRA EEGKQLARGM GVTEAKLQRW FKKRRVQFRR EH
Mouse|Rhox8|Rhox|PRD          LPRNRYRFTK FQLQELER-I FE-RNHYPSA AARRELARWI GVTESRVENW FKSRRAKYRK CL
Mouse|Rhox9|Rhox|PRD          QRTRRTRFTH SQLRDLER-L FQ-ENRFPSL RVRRDLARWM GVDESDVQEW FKMRRALFRR HS
Human|RHOXF1|Rhox|PRD         PRTRRTKFTL LQVEELES-V FR-HTQYPDV PTRRELAENL GVTEDKVRVW FKNKRARCRR HQ
Human|RHOXF2|Rhox|PRD         QQPNVHAFTP LQLQELER-I FQ-REQFPSE FLRRRLARSM NVTELAVQIW FENRRAKWRR HQ
Human|RHOXF2B|Rhox|PRD        QQPNVHAFTP LQLQELEC-I FQ-REQFPSE FLRRRLARSM NVTELAVQIW FENRRAKWRR HQ
Chicken|RX2|Rax|PRD           HRRNRTTFTT YQLHELER-A FE-KSHYPDV YSREELAMKV NLPEVRVQVW FQNRRAKWRR QE
Human|SEBOX|Sebox|PRD         HRRKRTTFSK GQLLELER-A FA-AWPYPNI STHEHLAWVT CLPEAKVQVW FQKRWAKIIK NR
Mouse|Sebox|Sebox|PRD         HRRKRTTFSV GQLVELER-V FA-ARPYPDI STREHLAQVT HLPEAKIQVW FQNRRAKRIK DR
Chicken|SEBOX|Sebox|PRD       -KRRRTTFSR GQLSELER-A FA-AVPYPDI ATRERLAELT QLPEAKIQVW FQNRRARRIR --
Human|SHOX|Shox|PRD           QRRSRTNFTL EQLNELER-L FD-ETHYPDA FMREELSQRL GLSEARVQVW FQNRRAKCRK QE
Frog|shox|Shox|PRD            QRRSRTNFTL EQLNELER-L FD-ETHYPDA FMREELSQRL GLSEARVQVW FQNRRAKCRK QE
Human|SHOX2|Shox|PRD          QRRSRTNFTL EQLNELER-L FD-ETHYPDA FMREELSQRL GLSEARVQVW FQNRRAKCRK QE
Mouse|Shox2|Shox|PRD          QRRSRTNFTL EQLNELER-L FD-ETHYPDA FMREELSQRL GLSEARVQVW FQNRRAKCRK QE
Chicken|SHOX2|Shox|PRD        QRRSRTNFTL EQLNELER-L FD-ETHYPDA FMREELSQRL GLSEARVQVW FQNRKAKCRK QE
Frog|shox2|Shox|PRD           QRRSRTNFTL EQLNELER-L FD-ETHYPDA FMREELSQRL GLSEARVQVW FQNRRAKCRK QE
Human|TPRX1|Tprx|PRD          QRQERTVYTE SQQKVLEF-Y FQ-KDQYPNY DQRLNLAEML SLREQQLQVW FKNRRAKLAR ER
Human|TPRXL|Tprx|PRD          QRQDRTIYNW KQQEVLEN-H FK-EEQYPDY DTRQELAEML NLREYQVQVW FKNRRAKRSR ER
Human|UNCX|Uncx|PRD           RRRTRTNFTG WQLEELEK-A FN-ESHYPDV FMREALALRL DLVESRVQVW FQNRRAKWRK KE
Mouse|Uncx|Uncx|PRD           RRRTRTNFTG WQLEELEK-A FN-ESHYPDV FMREALALRL DLVESRVQVW FQNRRAKWRK KE
Chicken|UNCX|Uncx|PRD         RRRTRTNFTG WQLEELEK-A FN-ESHYPDV FMREALALRL DLVESRVQV- ---------- --
Frog|uncx1|Uncx|PRD           QRRARANYSS WQLQELEK-A FQ-ATHYPDV FTREALALRL DLIEARVQVW FQNRRAKMRR QM
Frog|uncx2|Uncx|PRD           RRRTRTNFTG WQLEELEK-A FN-ESHYPDV FMREALALRL DLVESRVQVW FQNRRAKWRK KE
Human|VSX1|Vsx|PRD            KRRHRTVFTA HQLEELEK-A FS-EAHYPDV YAREMLAVKT ELPEDRIQVW FQNRRAKWRK RE
Mouse|Vsx1|Vsx|PRD            KRRHRTVFTA HQLEELEK-A FG-EAHYPDV YAREMLAAKT ELPEDRIQVW FQNRRAKWRK RE
Chicken|VSX1|Vsx|PRD          KRRHRTVFTA HQLEELEK-A FN-EAHYPDV YAREMLAVKT ELPEDRIQVW FQNRRAKWRK RE
Frog|vsx1|Vsx|PRD             KRRHRTVFTA HQLEELEK-A FN-EAHYPDV YAREMLALKT ELPEDRIQVW FQNRRAKWRK RE
Human|VSX2|Vsx|PRD            KRRHRTIFTS YQLEELEK-A FN-EAHYPDV YAREMLAMKT ELPEDRIQVW FQNRRAKWRK RE
Mouse|Vsx2|Vsx|PRD            KRRHRTIFTS YQLEELEK-A FN-EAHYPDV YAREMLAMKT ELPEDRIQVW FQNRRAKWRK RE
Chicken|VSX2|Vsx|PRD          KRRHRTIFTS YQLEELEK-A FN-EAHYPDV YAREMLAMKT ELPEDRIQVW FQNRRAKWRK RE
Frog|vsx2|Vsx|PRD             KRRHRTIFTS YQLEELEK-A FN-EAHYPDV YAREMLAMKT ELPEDRIQVW FQNRRAKWRK RE
OgarLEUTX                     GRRPRTTFSK EQLEELRL-V FT-TATHPDC ATIVNLAAKF CLEESVITTW FKNQRVRWRK EQ
NbenLEUTX1                    TRRPRTTFSK EQLEELRL-V FT-KAMHPDC TTIMNLAAKF CLDESVIMTW FKNQRVRWRK EH
NbenLEUTX2                    ISWHCTTFSL EQLAELRV-E FTEKTMSPDW VTIMSLTSRF HLDESVIMNH HPWSNGLHQD QQ
LcatLEUTX1                    RRRPRTQFTP EQLTVLEA-Q FT-KTTHPGW DAVQALASRL HLEESVIQTW FKNQCAKRRK LQ
LcatLEUTX2                    RRRPRTQFAP EQLRVLEA-Q FT-KTTHPGW DAVQALASRL HLEESVIQTW FKNQCAKRRK LQ
PsimLEUTX                     RRRPRTQFTP EQLRVLEA-Q FT-KTTHPGW DAVQALASRL HLEESVIQTW FKNQCAKRRK LQ
PcoqLEUTX                     HRRPRTSFSP EQLRVLEE-E FR-KTTHPGW DAVQALASRL HLDESVIKTW FKNQCAKWRK LR
CmedLEUTX                     RRRPRTSFSL EQLRVLEL-E FW-KTTHPDW DLVQALASRL HLDEVTIKTW FKNQRAKQRK LQ
MmurLEUTX2                    RRRPRTGFSA EQLRVLEE-E FR-KTTHPGW DEVQALAARL DLNETAIKTW FKNRRAKQRK LG
MmurLEUTX6                    RRRPRTGFSA EQLRVLEE-E FQ-KTTHPGW DEVQALAARL NLNETAIKTW FKNRRAKLRK LG
CsyrLEUTX                     ARRERTDFSP EQLGELRK-V FE-KTRTPNW VTIQELASKL HLDELVVKTW FKNQRAKRKK QL
PdonLEUTX                     DRRRRTTLFS QQLKALRK-M FE-ETMYPSL ATMVKLASEQ NLDLFVVKTW FKNQRAKWKK QQ
PpitLEUTX                     DRRRRTIFFS QQLKALRK-M FE-ETTYPSL TTMVKLASEQ HLDLSVVKNW FKNQRAKWKK QQ
ApalLEUTX                     DRRRRTTILS QQLKALRK-M FE-ETTYPSL ATMVKLASEQ HLDLCVVKTW FKNQRAKWKK QQ
AhybLEUTX                     DRRRRTIILP QQLKALRK-M FE-ETTHPSL ATMVKLASEQ HLDLSVVKTW FKNQRAKWKR QQ
AgeoLEUTX                     DRRRRTIILP QQLKALRK-M FE-ETTHPSL ATMVKLASEQ HLDLSVVKTW FKNQRAKWKR QQ
SapeLEUTX                     DRRRRTILLS EQLKALRK-M FE-ETTYPSL ATMVKLASEQ HLSLLVVKTW FKNRRAKWKK QQ
SbolLEUTX                     VRRHRTTFLS QQLMALRK-M FE-KTTYPSL ATMVTLASEQ QLNLPVVQTW FKNQRAKRKK QQ
AnanLEUTX                     DRRRRTILLS QQLKALRK-M FE-ETMYPSL AAMVTLASEQ HLDLSVVKTW FKNQRAKWKK QQ
CjacLEUTX                     DRRHRTKLLS HQLEALRK-T FE-ETMYPSL ATMVKLASEQ HLDLSVVKIW FKNQRAKWKQ QQ
SmidLEUTX                     DRRHRTKLLS HQLEALRK-V FE-ETMHPSL ATMVKLASEQ HLDLFVVKTW FKNQRAKWKK QQ
SimpLEUTX                     DRRHRTKLLS HQLEALRK-V FE-ETMHPSL ATMVKLASEQ HLDLFVVKTW FKNQRAKWKK QQ
PabeLEUTX                     DRRPRTRFLP KQLTALRE-L LE-KTMHPSL ATMGKLASKL QLDLSIVKTW FKNQRAKWKR QW
GgorLEUTX                     YRRPRTRFLS KQLTALRE-L LE-KTMHPSL ATMGKLASKI QLDLSVVKIW FKNQRAKWKR QQ
HsapLEUTX                     YRRPRTRFLS KQLTALRE-L LE-KTMHPSL ATMGKLASKL QLDLSVVKIW FKNQRAKWKR QQ
PtroLEUTX                     YRRPRTRFLS KQLTALRE-L LE-KTMHPSL ATMGKLASKL QLDLSVVKIW FKNQRAKWKR QQ
```

```
PpanLEUTX        YRRPRTRFLS KQLTALRE-L LE-KTMHPSL ATMGKLASKL QLDLSVVKIW FKNQRAKWKR QQ
HleuLEUTX        GRRPRTRFLS KQLAALRE-L LE-KTMHPSL ATMGKLASKL QLDLCVVKTW FKNQRAKWNR QR
NsikLEUTX        GRRPRTRFLS KQLAALRE-L LE-KTMHPSL ATMGKLASKL QLDLCVVKTW FKNQRAKWNR QR
NleuLEUTX        GRRPRTRFLS KQLAALRE-L LE-KTMHPSL ATMGKLASKL QLDLCVVKTW FKNQRAKWNR QR
SsynLEUTX        DRRPRTRFLS KQLTALRE-L LE-KTMHPSL ATMGKLASKL QLDLCVVKTW FKNQRAKWNR QR
HmolLEUTX        DRRPRTRFLS KQLAALRE-L LE-KTMHPSL ATMGKLASKL QLDLCVVKTW FKNQRAKWNR QR
HpilLEUTX        DRRPRTRFLS KQLAALRE-L LE-KTMHPSL ATMGKLASKL QLDLYVVKTW FKNQRAKWNR QR
PtepLEUTX        ERRQRTRFLS KQLTALTE-V LA-KTMHPSL VTMGKLASAL QLDLSVVKIW FKNQRAKWKR QQ
CangLEUTX        ERRQRTRFLS KQLTALRE-V LA-KTMHPSL VTMGKLASAL QLDLSVVKIW FKNQRAKWKR QQ
CgueLEUTX        ERRQRTRFLS KQLTALRE-V LA-KTMHPSL VTMGKLASAL QLDLSVVKIW FKNQRAKWKR QQ
TfraLEUTX        KRRQRTRFLS KQLTALRE-V LA-KTMHPSL VTMGKLASTL QLDLPVVKIW FKNQRAKWKR QQ
SentLEUTX        KRRQRTRFLS KQLTALRE-V LA-KTMHPSL VTMGKLASTL QLDLPVVKIW FKNQRAKWKR QQ
PnemLEUTX        KRRQRTRFLS KQLTALRE-V LA-KTMHPSL VTMGKLASTL QLDLSVVKIW FKNQRAKWKR QQ
NlarLEUTX        KRRQRTRFLS KQLTALRE-V LA-KTMHPSL VTMGKLASTL QLDLSVVKIW FKNQRAKWKR QQ
RbieLEUTX        KRRQRTRFLS KQLTALRE-V LA-KTMHPSL VTMGKLASTL QLDLSVVKIW FKNQRAKWKR QQ
RroxLEUTX        KRRQRTRFLS KQLTALRE-V LA-KTMHPSL VTMGKLASTL QLDLSVVKIW FKNQRAKWKR QQ
CmonLEUTX        ERRQRTRFLS KQLTALRE-V LA-KTMHPSL VTMGKLASTL QLDLSVVKIW FKNQRAKWKR QQ
CsabLEUTX        ERRQRTRFLS KQLTALRE-V LA-KTMHPSL VTMGKLASVL QLDLSVVKIW FKNQRAKWKR QQ
TgelLEUTX        ERRQRTRFLS KQLTALRE-V LA-KTMHPSL VTMGKLASTL QLDLSVVKIW FKNQRAKWKR QQ
PanuLEUTX        ERRQRTRFLS KQLTALRE-V LA-KTMHPSL VTMGKLASTL QLDLSVVKIW FKNQRAKWKK QQ
CatyLEUTX        ERRQRTRFLS KQLTALRE-V LA-KTMHPSL VTMGKLASTL QLDLSVVKIW FKNQRAKWKR QQ
MleuLEUTX        ERRQRTRFLS KQLTALRE-V LA-KTMHPSL VTMGKLASTL QLDLSVVKIW FKNQRAKWKR QQ
MsphLEUTX        ERRQRTRFLS KQLTALRE-V LA-KTMHPSL VTMGKLASTL QLDLSVVKIW FKNQRAKWKR QQ
MnigLEUTX        ERRQRTRFLS KQLTALRE-V LA-KTMHPSL VTMGKLASTL QLDLSVVKIW FKNQRAKWKR QQ
MarcLEUTX        ERRQRTRFLS KQLTALRE-V LA-KTMHPSL VTMGKLASTL QLDLSVVKIW FKNQRAKWKR QQ
MthiLEUTX        ERRQRTRFLS KQLTALRE-V LA-KTMHPSL VTMGKLASTL QLDLSVVKIW FKNQRAKWKR QQ
MfasLEUTX        ERRQRTRFLS KQLTALRE-V LA-KTMHPSL VTMGKLASTL QLDLSVVKIW FKNQRAKWKR QQ
MfusLEUTX        ERRQRTRFLS KQLTALRE-V LA-KTMHPSL VTMGKLASTL QLDLSVVKIW FKNQRAKWKR QQ
MmulLEUTX        ERRQRTRFLS KQLTALRE-V LA-KTMHPSL VTMGKLASTL QLDLSVVKIW FKNQRAKWKR QQ
SvulLEUTX        NRRQRTNFTP EQLVVLRD-A FQ-KNRSPNW ETIQELASLL NLEDIIVKTW FKNQRVRWKK QQ
BtauLEUTX        TRRFRTRFNG EQLGALRD-V FE-RTRYPHC FLIRTLASTI HLDASVIKTW FKNQRVKRRR EE
RferLEUTX        TRRYRTIFSP EQLGILKD-T FE-KTMYPHW VTMTALTSAT RLDELVIKTW FKNQRIKRRK QQ
EcabLEUTX        ARRCRTVFTP EQLRALKD-V FE-KTMYPDW FTITELTSSI DLEESVIKTW FKNQRVKRKK QQ
FcatLEUTX        ARRRRTHFSA EQLQALKQ-V FE-ETMYPDW VTMMELISST QLDESVIKTW FKNQRVKRKK EQ
```

Figure S2 Alignment of PRD-class homeodomains used as basis for tree in Figure S1. Species name abbreviations as in Figure 1 main text.

Figure S3

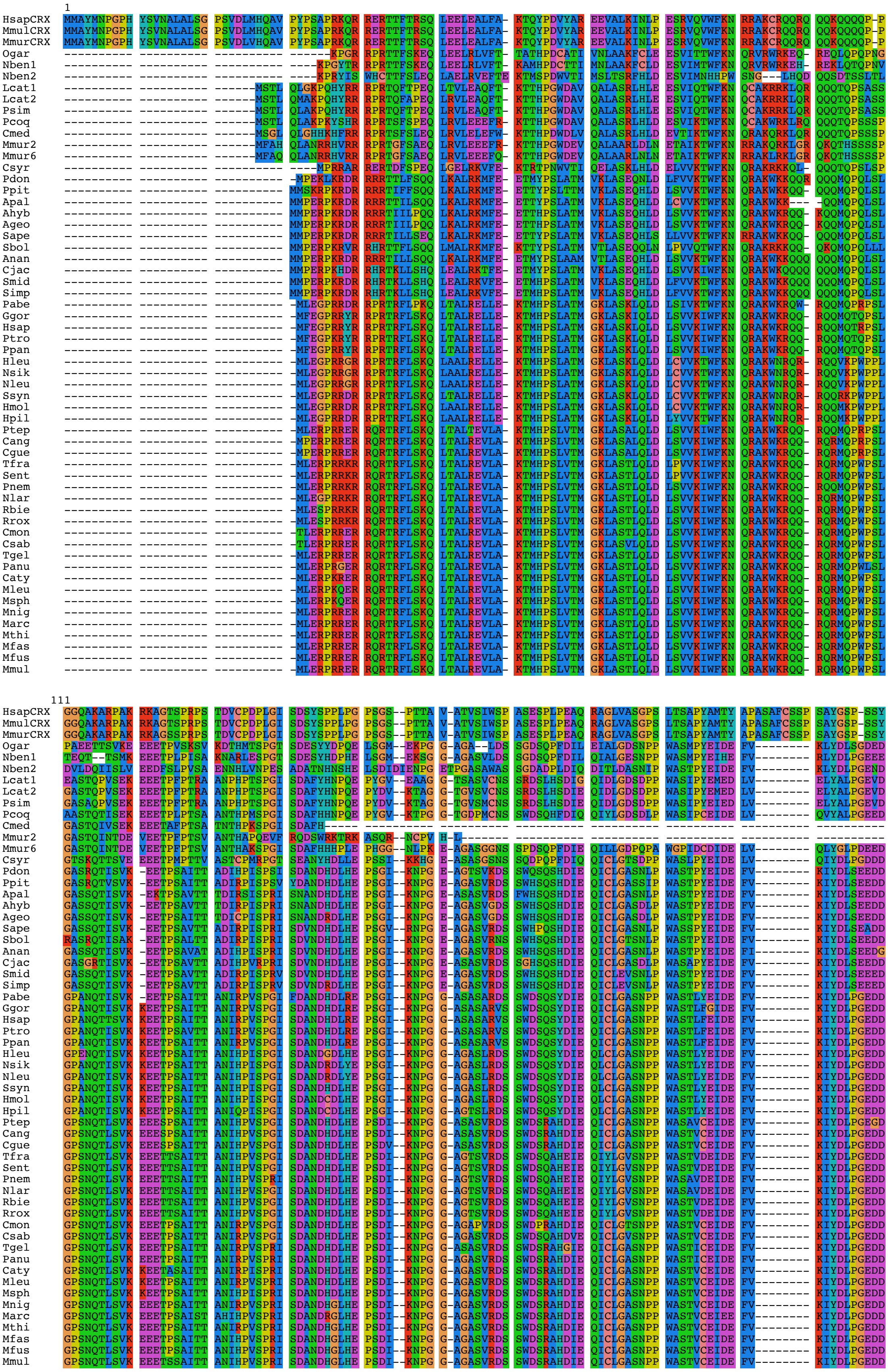
1
HsapCRX
MmulCRX
MmurCRX
Ogar
Nben1
Nben2
Lcat1
Lcat2
Psim
Pcoq
Cmed
Mmur2
Mmur6
Csyr
Pdon
Ppit
Apal
Ahyb
Ageo
Sape
Sbol
Anan
Cjac
Smid
Simp
Pabe
Ggor
Hsap
Ptro
Ppan
Hleu
Nsik
Nleu
Ssyn
Hmol
Hpil
Ptep
Cang
Cgue
Tfra
Sent
Pnem
Nlar
Rbie
Rrox
Cmon
Csab
Tgel
Panu
Caty
Mleu
Msph
Mnig
Marc
Mthi
Mfas
Mfus
Mmul
111

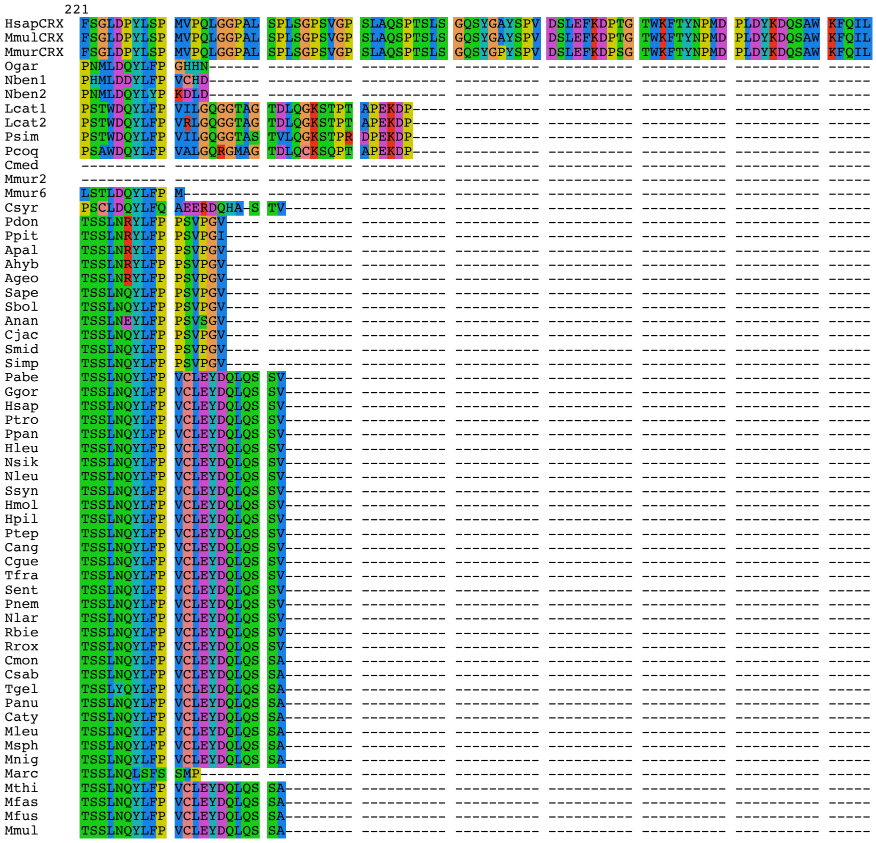

Figure S3 Alignment of LEUTX protein sequences. Alignment used for tree in main text Figure 1B. Species name abbreviations as in Figure 1 main text.

Figure S4

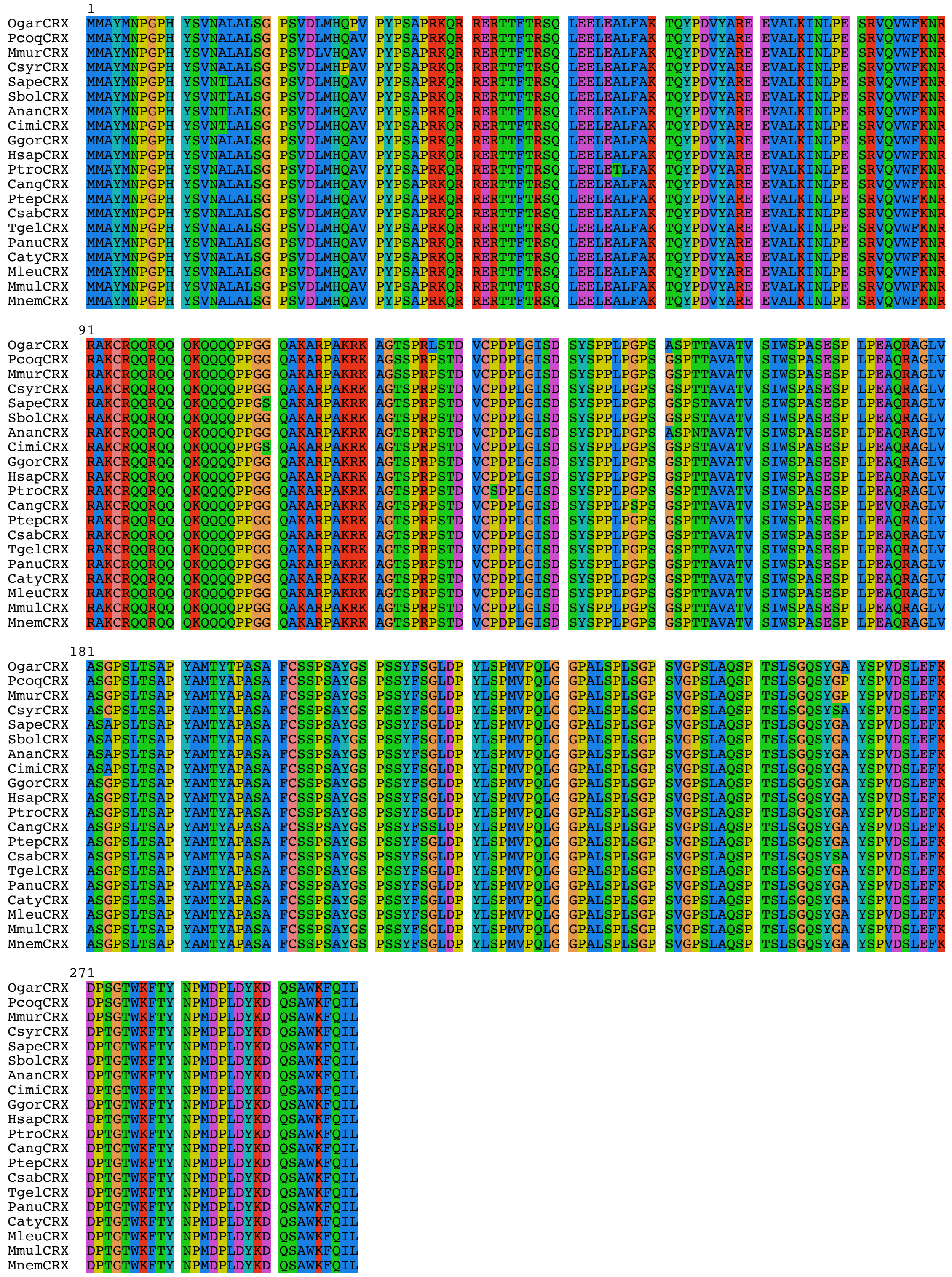

Figure S4 Alignment of primate CRX proteins. Species name abbreviations as in Figure 1 main text.

Figure S5

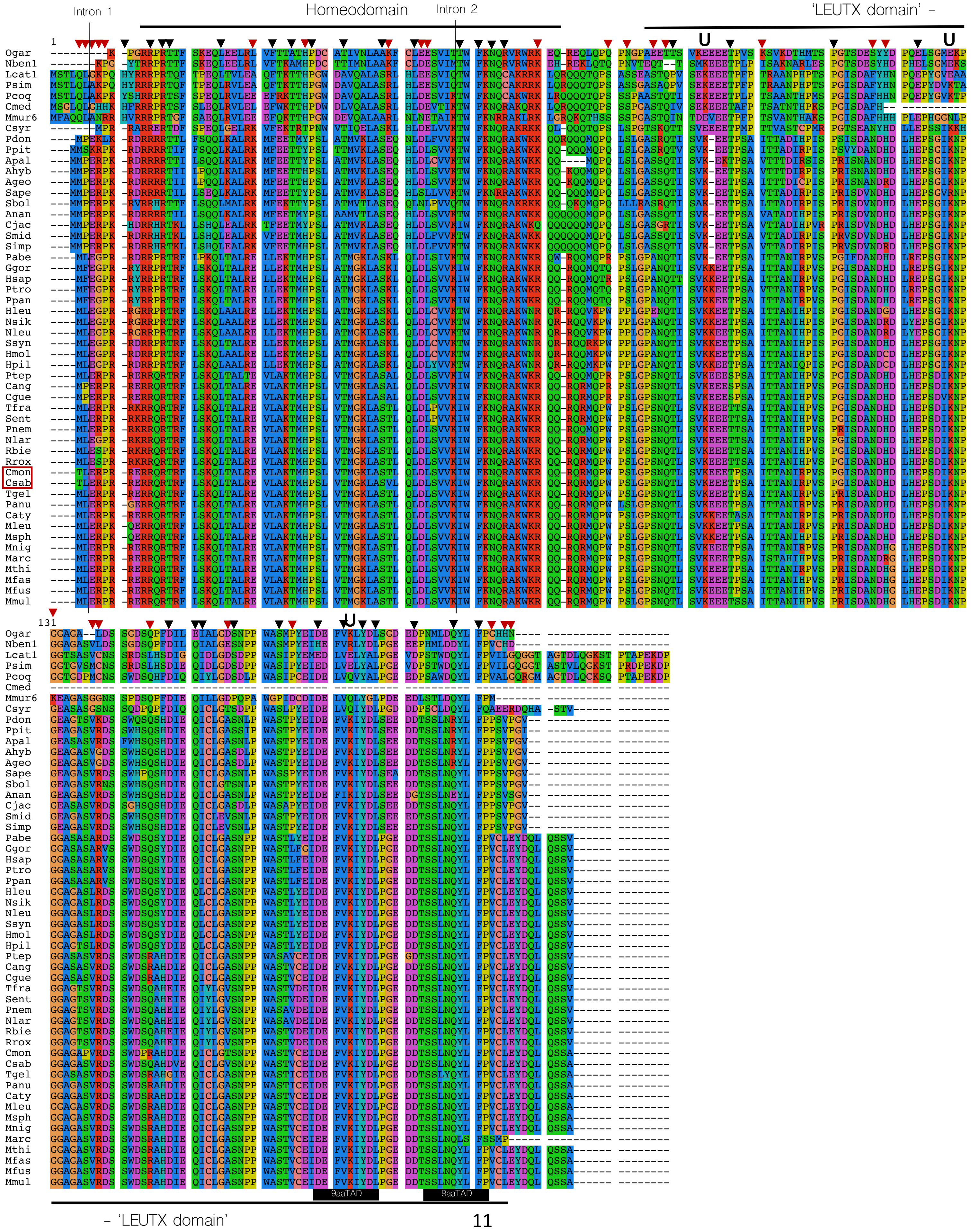
Intron 1
Homeodomain
Intron 2
'LEUTX domain' –
– 'LEUTX domain'
9aaTAD
9aaTAD
1
131

Figure S5 LEUTX protein sequence alignment used for tests for selection. Red arrowheads indicate residues identified as under positive selection; residues marked with a black arrowhead are under pervasive purifying selection. Human ubiquitination sites are marked with a U. The position of human 9aaTADs is marked with a labelled black bar. The Cmed sequence is shortened due to an incomplete assembly, not a premature stop codon. The red square marks Cmon and Csab, in which the New World monkey start codon has mutated to a threonine residue. Ogar and Nben lack a start codon because the first LEUTX exon could not be identified. Species name abbreviations as in Figure 1 main text.

Figure S6

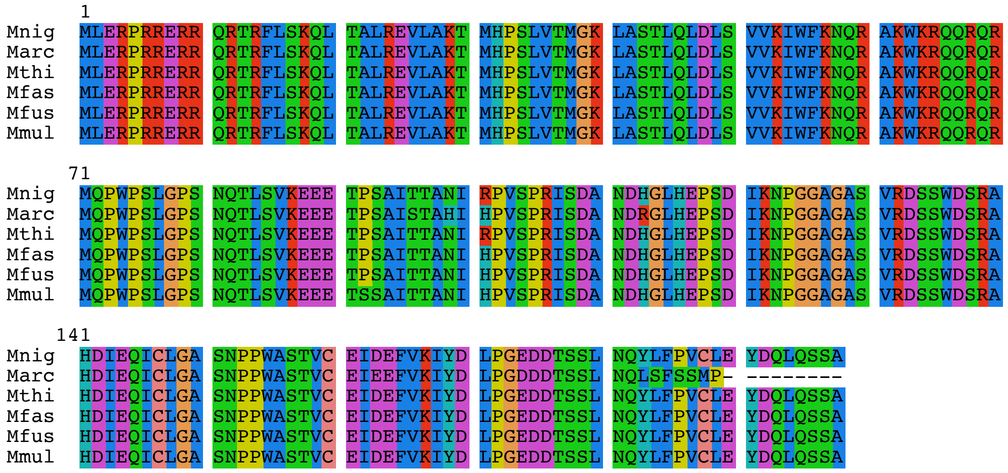

Figure S6 LEUTX proteins of species in the genus *Macaca*. Species name abbreviations as in Figure 1 main text.

Figure S7

>Ogar
exon 1 missing
KPGRRPRTTFSKEQLEELRLVFTTATHPDCATIVNLAAKFCLEESVITTWFKNQRVRWRKEQ
REQLQPQPNGPAEETTSVKEEEETPVSKSVKDTHMTSPGTSDESYYDPQELSGMEKPGGAGA
LDSSGDSQPFDILEIALGDSNPPWASMPYEIDEFVKLYDLSGDEDPNMLDQYLFPGHHN

>Nben1
exon 1 missing
KPGYTRRPRTTFSKEQLEELRLVFTKAMHPDCTTIMNLAAKFCLDESVIMTWFKNQRVRWRK
EHREKLQTQPNVTEQTTSMKEEETPLPISAKNARLESPGTSDESYHDPHELSGMEKSGGAGA
SVLDSSGDSQPFDILQIALGESNPPWASMPYEIHEFVRLYDLPGEEEPHMLDDYLFPVCHD

>Nben2
exon 1 missing
KPRYISWHCTTFSLEQLAELRVEFTEKTMSPDWVTIMSLTSRFHLDESVIMNHHPWSNGLHQ
DQQSDTSSLTLDVLDQIISLVEEDFSLPVSAENNHLVNPESADATNHNSHELSDIDIENPGE
TPGASAWASSGDADPLDIQDITLDASNIPWASTPYEIDEFVKLYDLPGENDPNMLDQYLYPK
DLD

>Lcat1
MSTLQLGKPQHYRRRPRTQFTPEQLTVLEAQFTKTTHPGWDAVQALASRLHLEESVIQTWFK
NQCAKRRKLQRQQQTQPSASSEASTQPVSEKEEETPFPTRAANPHPTSPGISDAFYHNPQEP
YGVEAAGGTSASVCNSSRDSLHSDIGQIDLGDSDPPWASIPYEMEDLVELYALPGEVDPSTW
DQYLFPVILGQGGTAGTDLQGKSTPTAPEKDP

>Lcat2
MSTLQMAKPQHYRRRPRTQFAPEQLRVLEAQFTKTTHPGWDAVQALASRLHLEESVIQTWFK
NQCAKRRKLQRQQQTQPSASSGASTQPVSEKEEETPFPTRAANPHPTSPGISDAFYHNPQEP
YDVKTAGGTGVSVCNSSRDSLHSDIEQIDLGDSDPPWASIPYEMEDLVELYALPGEVDPSTW
DQYLFPVRLGQGGTAGTDLQGKSTPTAPEKDP

>Psim
MSTLQLAKPQHYRRRPRTQFTPEQLRVLEAQFTKTTHPGWDAVQALASRLHLEESVIQTWFK
NQCAKRRKLQRQQQTQPSASSGASAQPVSEKEEETPFPTRAANPHPTSPGISDAFYHNPQEP
YDVKTAGGTGVSMCNSSRDSLHSDIEQIDLGDSDPPWASIPYEIEDLVELYALPGEVDPSTW
DQYLFPVILGQGGTASTVLQGKSTPRDPEKDP

>Pcoq
MSTLQLAKPKYSHRRPRTSFSPEQLRVLEEEFRKTTHPGWDAVQALASRLHLDESVIKTWFK
NQCAKWRKLRQQQQTQPSSSPAASTQTISEKEEETPLPTSAANTHPMSPGISDAFHHNPQEP
YGVKTPGGTGDPMCNSSWDSQHFDIQQIYLGDSDLPWASIPCEIDELVQVYALPGEEDPSAW
DQYLFPVALGQRGMAGTDLQCKSQPTAPEKDP

>Cmed
MSGLQLGHHKHFRRRPRTSFSLEQLRVLELEFWKTTHPDWDLVQALASRLHLDEVTIKTWFK
NQRAKQRKLQRQQQTQPSSSPGASTQIVSEKEEETAFPTSATNTHPKSPGISDAFH //
incomplete assembly, C-terminal domain missing.

>Mmur2
MFAHQLANRRHVRRRPRTGFSAEQLRVLEEEFRKTTHPGWDEVQALAARLDLNETAIKTWFK
NRRAKQRKLGRQKQTHSSSSPGASTQINTDEVEETPFPTSVANTHAPQEVFRQDSWRKTRKA
SQRNCPVHL

>Mmur6
MFAQQLANRRHVRRRPRTGFSAEQLRVLEEEFQKTTHPGWDEVQALAARLNLNETAIKTWFK
NRRAKLRKLGRQKQTHSSSSPGASTQINTDEVEETPFPTSVANTHAKSPGISDAFHHHPLEP
HGGNLPKEAGASGGNSSPDSQPFDIEQILLGDPQPAWGPIDCDIDELVQLYGLPDEEDLSTL
DQYLFPM

>Csyr
exon 1 missing
MPRRARRERTDFSPEQLGELRKVFEKTRTPNWVTIQELASKLHLDELVVKTWFKNQRAKRKK
QLQQQTQPSLSPGTSKQTTSVEEEETPMPTTVASTCPMRPGTSEANYHDLLEPSSIKKHGEA
SASGSNSSQDPQPFDIQQICLGTSDPPWASLPYEIDELVQIYDLPGDDDPSCLDQYLFQAEE
RDQHASTV

>Pdon
MPEKLKRDRRRRTTLFSQQLKALRKMFEETMYPSLATMVKLASEQNLDLFVVKTWFKNQRAK
WKKQQRQQQMQPQLSLGASRQTISVKEETPSAITTADIHPISPSISDANDHDLHEPSGIKNP
GEAGTSVKDSSWQSQSHDIEQICLGASNLPWASTPYEIDEFVKIYDLSEEDDTSSLNRYLFP
PSVPGV

>Ppit
MMSKRPKRDRRRRTIFFSQQLKALRKMFEETTYPSLTTMVKLASEQHLDLSVVKTWFKNQRA
KWKKQQQQQMQPQLSLGASRQTVSVKEETPSAITTADIRPISPSVYDANDHDLHEPSGIKNP
GEAGASVRDSSWHSQSHDIEQICLGASSIPWASTPYEIDEFVKIYDLSEEDDTSSLNRYLFP
PSVPGI

>Apal
MMPERPKRDRRRRTTILSQQLKALRKMFEETTYPSLATMVKLASEQHLDLCVVKTWFKNQRA
KWKKQQMQPQLSLGASSQTISVKEKTPSAVTTTDIRSISPRISNANDHDLHEPSGIKNPGEA
SASVRDSFWHSQSHDIEQICLGASNLPWASTPYEIDEFVKIYDLSEEDDTSSLNRYLFPPSV
PGV

>Ahyb
MMPERPKRDRRRRTIILPQQLKALRKMFEETTHPSLATMVKLASEQHLDLSVVKTWFKNQRA
KWKRQQKQQMQPQLSLGASSQTISVKEETPSAVTTTDIRPISPRISNANDHDLHEPSGIKNP
GEAGASVGDSSWHSQSHDIEQICLGASDLPWASTPYEIDEFVKIYDLSEEDDTSSLNRYLFP
PSVPGV

>Ageo
MMPERPKRDRRRRTIILPQQLKALRKMFEETTHPSLATMVKLASEQHLDLSVVKTWFKNQRA
KWKRQQKQQMQPQLSLGASSQTISVKEETPSAITTTDICPISPRISNANDRDLHEPSGIKNP
GEAGASVGDSSWHSQSHDIEQICLGASDLPWASTPYEIDEFVKIYDLSEEDDTSSLNRYLFP
PSVPGV

>Sape
MMPERPKRDRRRRTILLSEQLKALRKMFEETTYPSLATMVKLASEQHLSLLVVKTWFKNRRA
KWKKQQQQQMQPQLSLGASSQTISVKEETPSAVTTADIRPISPRISDVNDHDLHEPSGVRNP
GEAGASVRDSSWHPQSHDIEQICLGASNLPWASSPYEIDEFVKIYDLSEADDTSSLNQYLFP
PSVPGV

>Sbol
MMPERPKRVRRHRTTFLSQQLMALRKMFEKTTYPSLATMVTLASEQQLNLPVVQTWFKNQRA
KRKKQQQKQMQPQLLLRASRQTISAKEETPSALTTADIRPISPRISDVNDHDLHEPSGIKNP
GEAGASVRNSSWHSQSHDIEQICLGTSNLPWASTPYEIDEFVKIYDLSEEDDTSSLNQYLFP
PSVPGV

>Anan
MMPERPKRDRRRRTILLSQQLKALRKMFEETMYPSLAAMVTLASEQHLDLSVVKTWFKNQRA
KWKKQQQQQQMQPQLSLGASSQTISVKEETPSAVATADIHPISPRISDVNDHDLHEPSGIKN
PGEAGASVRDSSWHSQSHDIEQICLGASNLPWASTPYEIDEFIKIYDLSEEDGTSSLNEYLF
PPSVSGV

>Cjac
MMPERPKHDRRHRTKLLSHQLEALRKTFEETMYPSLATMVKLASEQHLDLSVVKIWFKNQRA
KWKQQQQQQQMQPQLSLGASGRTISVKEETPSAVTTADIHPVRPRISDVNDHDLHEPSGIKN
PGEASASVRDSSGHSQSHDIEQICLGASDLPWASAPYEIDEFVKIYDLSEEDDTSSLNQYLF
PPSVPGV

>Smid
MMPERPKRDRRHRTKLLSHQLEALRKVFEETMHPSLATMVKLASEQHLDLFVVKTWFKNQRA
KWKKQQQQQQMQPQLSLGASSQTISVKEETPSAVTTADIRPISPRVSDVNDHDLHEPSGIKN
PGEAGASVRDSSWHSQSHDIEQICLEVSNLPWASTPYEIDEFVKIYDLSEEEDTSSLNQYLF
PPSVPGV

>Simp
MMPERPKRDRRHRTKLLSHQLEALRKVFEETMHPSLATMVKLASEQHLDLFVVKTWFKNQRA
KWKKQQQQQQMQPQLSLGASSQTISVKEETPSAVTTADIRPISPRVSDVNDRDLHEPSGIKN
PGEAGASVRDSSWHSQSHDIEQICLEVSNLPWASTPYEIDEFVKIYDLSEEEDTSSLNQYLF
PPSVPGV

>Pabe
MLEGPRRDRRPRTRFLPKQLTALRELLEKTMHPSLATMGKLASKLQLDLSIVKTWFKNQRAK
WKRQWRQQMQPRPSLGPANQTISVKEETPSAITTANIRPVSPGIFDANDHDLREPSGIKNPG
GASASARDSSWDSQSYDIEQICLGASNPPWASTLYEIDEFVKIYDLPGEDDTSSLNQYLFPV
CLEYDQLQSSV

>Ggor
MFEGPRRYRRPRTRFLSKQLTALRELLEKTMHPSLATMGKLASKIQLDLSVVKIWFKNQRAK
WKRQQRQQMQTQPSLGPANQTISVKKEETPSAITTANIRPVSPGISDANDHDLREPSGIKNP
GGASASARVSSWDSQSYDIEQICLGASNPPWASTLFGIDEFVKIYDLPGEDDTSSLNQYLFP
VCLEYDQLQSSV

>Hsap
MFEGPRRYRRPRTRFLSKQLTALRELLEKTMHPSLATMGKLASKLQLDLSVVKIWFKNQRAK
WKRQQRQQMQTRPSLGPANQTTSVKKEETPSAITTANIRPVSPGISDANDHDLREPSGIKNP
GGASASARVSSWDSQSYDIEQICLGASNPPWASTLFEIDEFVKIYDLPGEDDTSSLNQYLFP
VCLEYDQLQSSV

>Ptro
MFEGPKRYRRPRTRFLSKQLTALRELLEKTMHPSLATMGKLASKLQLDLSVVKIWFKNQRAK
WKRQQRQQMQTQPSLGPANQTISVKKEETPSAVTTANIRPVSPGISDANDHDLREPSGIKNP
GGASASARVSSWDSQSYDIEQICLGASNPPWASTLFEIDEFVKIYDLPGEDDTSSLNQYLFP
VCLEYDQLQSSV

>Ppan
MFEGPRRYRRPRTRFLSKQLTALRELLEKTMHPSLATMGKLASKLQLDLSVVKIWFKNQRAK
WKRQQRQQMQTQPSLGPANQTISVKKEETPSAITTANIRPVSPGISDANDHDLREPSGIKNP
GGASASARVSSWDSQSYDIEQICLGASNPPWASTLFEIDEFVKIYDLPGEDDTSSLNQYLFP
VCLEYDQLQSSV

>Hleu
MLEGPRRGRRPRTRFLSKQLAALRELLEKTMHPSLATMGKLASKLQLDLCVVKTWFKNQRAK
WNRQRRQQVKPWPPLGPENQTISVKKEETPSAITTANIHPISPGISDANDGDLHEPSGIKNP
GGAGASLRDSSWDSQSYDIEQLCLGASNPPWASTLYEIDEFVKIYDLPGEDDTSSLNQYLFP
VCLEYDQLQSSV

>Nsik
MLEGPRRGRRPRTRFLSKQLAALRELLEKTMHPSLATMGKLASKLQLDLCVVKTWFKNQRAK
WNRQRRQQVKPWPPLGPANQTISVKKEETPSAITTANIHPISPGISDANDRDLYEPSGIKNP
GGAGASLRDSSWDSQSYDIEQLCLGASNPPWASTLYEIDEFVKIYDLPGEDDTSSLNQYLFP
VCLEYDQLQSSV

>Nleu
MLEGPRRGRRPRTRFLSKQLAALRELLEKTMHPSLATMGKLASKLQLDLCVVKTWFKNQRAK
WNRQRRQQVKPWPPLGPANQTISVKKEETPSAITTANIHPISPGISDANDRDLYEPSGIKNP
GGAGASLRDSSWDSQSYDIEQLCLGASNPPWASTLYEIDEFVKIYDLPGEDDTSSLNQYLFP
VCLEYDQLQSSV

>Ssyn
MLEGPRRDRRPRTRFLSKQLTALRELLEKTMHPSLATMGKLASKLQLDLCVVKTWFKNQRAK
WNRQRRQQRKPWPPLGPANQTISVKKEETPSAITTANIHPISPGISDANDHDLHEPSGIKNP
GGAGASLRDSSWDSQSYDIEQLCLGASNPPWASTLYEIDEFVKIYDLPGEDDTSSLNQYLFP
VCLEYDQLQSSV

>Hmol
MLEGPRRDRRPRTRFLSKQLAALRELLEKTMHPSLATMGKLASKLQLDLCVVKTWFKNQRAK
WNRQRRQQMKPWPPLGPANQTISVKKEETPSAITTANIHPISPGISDANDCDLHEPSGIKNP
GGAGASLRDSSWDSQSYDIEQLCLGASNPPWASTLYEIDEFVKIYDLPGEDDTSSLNQYLFP
VCLEYDQLQSSV

>Hpil
MLEGPRRDRRPRTRFLSKQLAALRELLEKTMHPSLATMGKLASKLQLDLYVVKTWFKNQRAK
WNRQRRQQMKPWPPLGPANQTISVKKEETPSAITTANIQPISPGISDANDCDLHEPSGIKNP
GGAGTSLRDSSWDSQSYDIEQLCLGASNPPWASTLYEIDEFVKIYDLPGEDDTSSLNQYLFP
VCLEYDQLQSSV

>Ptep
MLERPRRERRQRTRFLSKQLTALTEVLAKTMHPSLVTMGKLASALQLDLSVVKIWFKNQRAK
WKRQQRQQMQPRPSLGPSNQTLSVKEEESPSAITTANIHPVSPGISDANDHDLHEPSDIKNP
GGASASVRDSSWDSRAHDIEQICLGASNPPWASAVCEIDEFVKIYDLPGEGDTSSLNQYLFP
VCLEYDQLQSSV

>Cang
MPERPRRERRQRTRFLSKQLTALREVLAKTMHPSLVTMGKLASALQLDLSVVKIWFKNQRAK
WKRQQRQRMQPRPSLGPSNQTLSVKEEESPSAITTANIHPVSPGISDANDHDLHEPSDIKNP
GGASASVRDSSWDSRAHDIEQICLGASNPPWASTVCEIDEFVKIYDLPGEDDTSSLNQYLFP
VCLEYDQLQSSV

>Cgue
MPERPRRERRQRTRFLSKQLTALREVLAKTMHPSLVTMGKLASALQLDLSVVKIWFKNQRAK
WKRQQRQRMQPRPSLGPSNQTLSVKEEESPSAITTANIHPVSPGISDANDHDLHEPSDVKNP
GGASASVRDSSWDSRAHDIEQICLGASNPPWASTVCEIDEFVKIYDLPGEDDTSSLNQYLFP
VCLEYDQLQSSV

>Tfra
MLERPRRKRRQRTRFLSKQLTALREVLAKTMHPSLVTMGKLASTLQLDLPVVKIWFKNQRAK
WKRQQRQRMQPWPSLGPSNQTLSVKEEETTSAITTANIHPVSPGISDANDHDLHEPSDIKNP
GGAGTSVRDSSWDSQAHEIEQIYLGVSNPPWASTVDEIDEFVKIYDLPGEDDTSSLNQYLFP
VCLEYDQLQSSV

>Sent
MLERPRRKRRQRTRFLSKQLTALREVLAKTMHPSLVTMGKLASTLQLDLPVVKIWFKNQRAK
WKRQQRQRMQPWPSLGPSNQTLSVKEEETTSAITTANIHPVSPGISDANDHDLHEPSDIKNP
GGAGTSVRDSSWDSQAHEIEQIYLGVSNPPWASTVDEIDEFVKIYDLPGEDDTSSLNQYLFP
VCLEYDQLQSSV

>Pnem
MLERPRRKRRQRTRFLSKQLTALREVLAKTMHPSLVTMGKLASTLQLDLSVVKIWFKNQRAK
WKRQQRQQMQPWPSLGPSNQTLSVKEEETTSAITTANIHPVSPRISDANDHDLHEPSDIKNP
GGAGTSVRDSSWDSQAHEIEQIYLGVSNPPWASAVDEIDEFVKIYDLPGEDDTSSLNQYLFP
VCLEYDQLQSSV

>Nlar
MLEGPRRKRRQRTRFLSKQLTALREVLAKTMHPSLVTMGKLASTLQLDLSVVKIWFKNQRAK
WKRQQRQRMQPWPSLGPSNQTLSVKEEETTSAITTANIHPVSPGISDANDHDLHEPSDIKNP
GGAGTSVRDSSWDSQAHEIEQIYLGVSNPPWASAVDEIDEFVKIYDLPGEDDTSSLNQYLFP
VCLEYDQLQSSV

>Rbie
MLESPRRKRRQRTRFLSKQLTALREVLAKTMHPSLVTMGKLASTLQLDLSVVKIWFKNQRAK
WKRQQRQRMQPWPSLGPSNQTLSVKEEETTSAITTANIHPVSPGISDANDHDLHEPSDIKNP
GGAGTSVRDSSWDSQAHEIEQIYLGVSNPPWASTVDEIDEFVKIYDLPGEDDTSSLNQYLFP
VCLEYDQLQSSV

>Rrox
MLESPRRKRRQRTRFLSKQLTALREVLAKTMHPSLVTMGKLASTLQLDLSVVKIWFKNQRAK
WKRQQRQRMQPWPSLGPSNQTLSVKEEETTSAITTANIHPVSPGISDANDHDLHEPSDIKNP
GGAGTSVRDSSWDSQAHEIEQIYLGVSNPPWASTVDEIDEFVKIYDLPGEDDTSSLNQYLFP
VCLEYDQLQSSV

>Cmon
TLERPRRERRQRTRFLSKQLTALREVLAKTMHPSLVTMGKLASTLQLDLSVVKIWFKNQRAK
WKRQQRQRMQPWPSLGPSNQTLSVKEEETPSAITTANIRPVSPGISDANDHDLHEPSDIKNP
GGAGAPVRDSSWDPRAHDIEQICLGTSNPPWASTVCEIDEFVKIYDLPGEDDTSSLNQYLFP
VCLEYDQLQSSA

>Csab
TLERPRRERRQRTRFLSKQLTALREVLAKTMHPSLVTMGKLASVLQLDLSVVKIWFKNQRAK
WKRQQRQRMQPWPSLGPSNQTLSVKEEETPSAITTANIRPVSPGISDANDHDLHEPSDIKNP
GGAGASVRDSSWDSQAHDVEQICLGVSNPPWASTVCEIDEFVKIYDLPGEDDTSSLNQYLFP
VCLEYDQLQSSA

>Tgel
MLERPRRERRQRTRFLSKQLTALREVLAKTMHPSLVTMGKLASTLQLDLSVVKIWFKNQRAK
WKRQQRQRMQPWPSLGPSNQTLSVKEEETPSAITTANIRPVSPRISDANDHDLHEPSDIKNP
GGASASVRDSSWDSRAHGIEQICLGASNPPWASTICEIDEFVKIYDLPGEDDTSSLYQYLFP
VCLEYDQLQSSA

>Panu
MLERPRGERRQRTRFLSKQLTALREVLAKTMHPSLVTMGKLASTLQLDLSVVKIWFKNQRAK
WKKQQRQRMQPWLSLGPSNQTLSVKEEETPSAITTANIRPVSPRISDANDHDLHEPSDIKNP
GGAGASVRDSSWDSRAHDIEQICLGASNPPWASTICEIDEFVKIYDLPGEDDTSSLNQYLFP
VCLEYDQLQSSA

>Caty
MLERPKRERRQRTRFLSKQLTALREVLAKTMHPSLVTMGKLASTLQLDLSVVKIWFKNQRAK
WKRQQRQRMQPWPSLGPSNQTLSVKKEETASAITTANIRPISPRISDANDHDLHEPSDIKNP
GGAGASVRDSSWDSRAHDIEQICLGASNPPWASTVCEIDEFVKIYDLPGEDDTSSLNQYLFP
VCLEYDQLQSSA

>Mleu
MLERPKQERRQRTRFLSKQLTALREVLAKTMHPSLVTMGKLASTLQLDLSVVKIWFKNQRAK
WKRQQRQRMQPWPSLGPSNQTLSVKKEETPSAITTANIRPVSPRISDANDHDLHEPSDIKNP
GGAGASVRDSSWDSRAHDIEQICLGASNPPWASTVCEIDEFVKIYDLPGEDDTSSLNQYLFP
VCLEYDQLQSSA

>Msph
MLERPKQERRQRTRFLSKQLTALREVLAKTMHPSLVTMGKLASTLQLDLSVVKIWFKNQRAK
WKRQQRQRMQPWPSLGPSNQTLSVKKEETPSAITTANIHPISPRISDANDHDLHEPSDIKNP
GGAGASVRDSSWDSRAHDIEQICLGASNPPWASTVCEIDEFVKIYDLPGEDDTSSLNQYLFP
VCLEYDQLQSSA

>Mnig
MLERPRRERRQRTRFLSKQLTALREVLAKTMHPSLVTMGKLASTLQLDLSVVKIWFKNQRAK
WKRQQRQRMQPWPSLGPSNQTLSVKEEETPSAITTANIRPVSPRISDANDHGLHEPSDIKNP
GGAGASVRDSSWDSRAHDIEQICLGASNPPWASTVCEIDEFVKIYDLPGEDDTSSLNQYLFP
VCLEYDQLQSSA

>Marc
MLERPRRERRQRTRFLSKQLTALREVLAKTMHPSLVTMGKLASTLQLDLSVVKIWFKNQRAK
WKRQQRQRMQPWPSLGPSNQTLSVKEEETPSAISTAHIHPVSPRISDANDRGLHEPSDIKNP
GGAGASVRDSSWDSRAHDIEQICLGASNPPWASTVCEIEEFVKIYDLPGDDDTSSLNQLSFS
SMP

>Mthi
MLERPRRERRQRTRFLSKQLTALREVLAKTMHPSLVTMGKLASTLQLDLSVVKIWFKNQRAK
WKRQQRQRMQPWPSLGPSNQTLSVKEEETPSAITTANIRPVSPRISDANDHGLHEPSDIKNP
GGAGASVRDSSWDSRAHDIEQICLGASNPPWASTVCEIDEFVKIYDLPGEDDTSSLNQYLFP
VCLEYDQLQSSA

>Mfas
MLERPRRERRQRTRFLSKQLTALREVLAKTMHPSLVTMGKLASTLQLDLSVVKIWFKNQRAK
WKRQQRQRMQPWPSLGPSNQTLSVKEEETPSAITTANIHPVSPRISDANDHGLHEPSDIKNP
GGAGASVRDSSWDSRAHDIEQICLGASNPPWASTVCEIDEFVKIYDLPGEDDTSSLNQYLFP
VCLEYDQLQSSA

>Mfus
MLERPRRERRQRTRFLSKQLTALREVLAKTMHPSLVTMGKLASTLQLDLSVVKIWFKNQRAK
WKRQQRQRMQPWPSLGPSNQTLSVKEEETPSAITTANIHPVSPRISDANDHGLHEPSDIKNP
GGAGASVRDSSWDSRAHDIEQICLGASNPPWASTVCEIDEFVKIYDLPGEDDTSSLNQYLFP
VCLEYDQLQSSA

>Mmul
MLERPRRERRQRTRFLSKQLTALREVLAKTMHPSLVTMGKLASTLQLDLSVVKIWFKNQRAK
WKRQQRQRMQPWPSLGPSNQTLSVKEEETSSAITTANIHPVSPRISDANDHGLHEPSDIKNP
GGAGASVRDSSWDSRAHDIEQICLGASNPPWASTVCEIDEFVKIYDLPGEDDTSSLNQYLFP
VCLEYDQLQSSA

Figure S7 Primate LEUTX sequences. LEUTX protein sequences annotated in this work.

Figure S8

```
>Hsap_LEUTX_V5
ATGTTTGAGGGCCCCAGAAGATACAGACGGCCCAGAACCAGATTCCTGAGCAAGCAGCTGACAGCCCT
GAGAGAGCTGCTGGAAAAGACAATGCACCCCAGCCTGGCCACCATGGGAAAGCTGGCTTCTAAACTGC
AGCTGGACCTGAGCGTGGTCAAGATCTGGTTCAAGAACCAGCGGGCCAAGTGGAAGCGGCAGCAGAGA
CAGCAGATGCAGACCAGACCTTCTCTGGGCCCTGCCAATCAGACCACCAGCGTGAAGAAAGAGGAAAC
CCCTAGCGCCATCACCACCGCCAACATCAGACCTGTGTCTCCCGGCATCAGCGACGCCAACGATCACG
ATCTGAGAGAACCCAGCGGCATCAAGAATCCTGGCGGAGCCTCTGCCTCTGCCAGAGTGTCATCTTGG
GACAGCCAGAGCTACGACATCGAGCAGATCTGTCTGGGCGCCAGCAATCCTCCTTGGGCCAGCACACT
GTTCGAGATCGACGAGTTCGTGAAGATCTACGACCTGCCTGGCGAGGACGATACCAGCAGCCTGAACC
AGTATCTGTTCCCCGTGTGCCTGGAATACGATCAGCTGCAGAGTTCTGTTGGCGGCGGAGGATCTGGC
GGAGGCGGTTCTGGAAAGCCCATTCCTAATCCTCTGCTGGGCCTCGACAGCACCTGATGATAA


>Cjac_LEUTX_V5
ATGATGCCCGAAAGACCCAAGCACGACAGACGGCACAGAACAAAGCTGCTGAGCCACCAGCTGGAAGC
CCTGAGAAAGACCTTCGAGGAAACAATGTACCCCAGCCTGGCCACCATGGTCAAGCTGGCCTCTGAAC
AGCACCTGGACCTGAGCGTGGTCAAGATCTGGTTCAAGAACCAGCGGGCCAAGTGGAAGCAGCAGCAA
CAGCAACAACAGATGCAGCCCCAGCTGTCTCTGGGCGCCTCTGGCAGAACAATCAGCGTGAAAGAAGA
GACACCCAGCGCCGTGACCACCGCCGATATTCACCCTGTGCGGCCTAGAATCAGCGACGTGAACGACC
ACGATCTGCACGAGCCTAGCGGCATCAAGAATCCTGGCGAAGCCTCTGCCAGCGTGCGGGATTCTTCT
GGCCACAGCCAGAGCCACGACATCGAGCAGATTTGTCTGGGAGCCAGCGACCTGCCTTGGGCCTCTGC
TCCTTATGAGATCGACGAGTTCGTGAAGATCTACGACCTGTCCGAAGAGGACGACACCAGCAGCCTGA
ACCAGTACCTGTTTCCTCCAAGCGTGCCAGGCGTTGGAGGCGGAGGATCTGGCGGAGGCGGATCTGGA
AAGCCCATTCCTAATCCTCTGCTGGGCCTCGACTCCACCTGATGATAA
```

Figure S8 *LEUTX* expression constructs. Sequences of ectopically expressed *LEUTX* genes, including GGGGSGGGGS linkers (blue) and V5 tag (red). Abbreviations: Cjac = *Callithrix jacchus*; Hsap = *Homo sapiens*.
